# MCR Scaffolds Get Hotter with ^18^F-Labeling

**DOI:** 10.3390/molecules24071327

**Published:** 2019-04-04

**Authors:** Tryfon Zarganes-Tzitzikas, Gonçalo S. Clemente, Philip H. Elsinga, Alexander Dömling

**Affiliations:** 1 Department of Drug Design, Groningen Research Institute of Pharmacy, University of Groningen, 9713 AV Groningen, The Netherlands; ttzitzikas@yahoo.com; 2 Department of Nuclear Medicine and Molecular Imaging, University Medical Center Groningen, University of Groningen, 9713 GZ Groningen, The Netherlands; g.dos.santos.clemente@umcg.nl

**Keywords:** multicomponent reactions, fluor-18, positron emission tomography, radiochemistry, boronic pinacol esters

## Abstract

Imaging techniques, such as positron emission tomography (PET), represent great progress in the clinical development of drugs and diagnostics. However, the efficient and timely synthesis of appropriately labeled compounds is a largely unsolved problem. Numerous small drug-like molecules with high structural diversity can be synthesized via convergent multicomponent reactions (MCRs). The combination of PET labeling with MCR synthesis of biologically active compounds can greatly simplify radioanalytical and imaging-based analysis. In a proof-of-concept study, we optimized robust on-site radiolabeling conditions that were subsequently applied to several structurally different drug-like MCR scaffolds (e.g., arenes, β-lactam, tetrazole, and oxazole). These labeled scaffolds were synthesized via pinacol-derived aryl boronic esters (arylBPin) by copper-mediated oxidative ^18^F-fluorination with radiochemical conversions (RCCs) from 15% to 76%.

## 1. Introduction

Recently, the pharmaceutical industry has demonstrated an increasing interest in accelerating data acquisition using radioanalytical and translational molecular imaging strategies during the early stages of drug discovery. Molecular imaging uses specific tracers to study cellular or subcellular processes, ideally without intervening in them or causing a biological response. In the medical field, it has already shown to have a major impact on health care systems by providing longitudinal studies with three-dimensional and quantitative images. This facilitates the diagnosis of a wide range of pathologies and the assessment to treatment response, bringing personalized therapy into routine clinical practice. The unique capacity of positron emission tomography (PET) techniques to detect with high sensitivity (10^−12^ mol/L) nano- to picomolar amounts of analyte, clearly benefits from efficient use of resources and raised the demand for radiotracers and radiolabeling techniques. In the clinical field, PET imaging is widely used mainly due to a nonspecific radiotracer analog of glucose—2-[^18^F]fluoro-2-deoxy-glucose ([^18^F]FDG)—that can identify changes in cellular glucose metabolism during inflammatory processes or related to the hypermetabolism of tumor cells. Radioanalytical and nuclear imaging techniques can also significantly shorten the temporal gap between preclinical research and first-in-human clinical trials by facilitating study design and further submission for regulatory agency approval [1,2,3]. PET applications range from the assessment and mapping of potential therapeutic targets to the characterization, validation, and evaluation of toxicology, pharmacokinetic, and pharmacodynamics. However, the efficient and timely synthesis of appropriately labeled compounds is a major issue.

**Figure 1:**
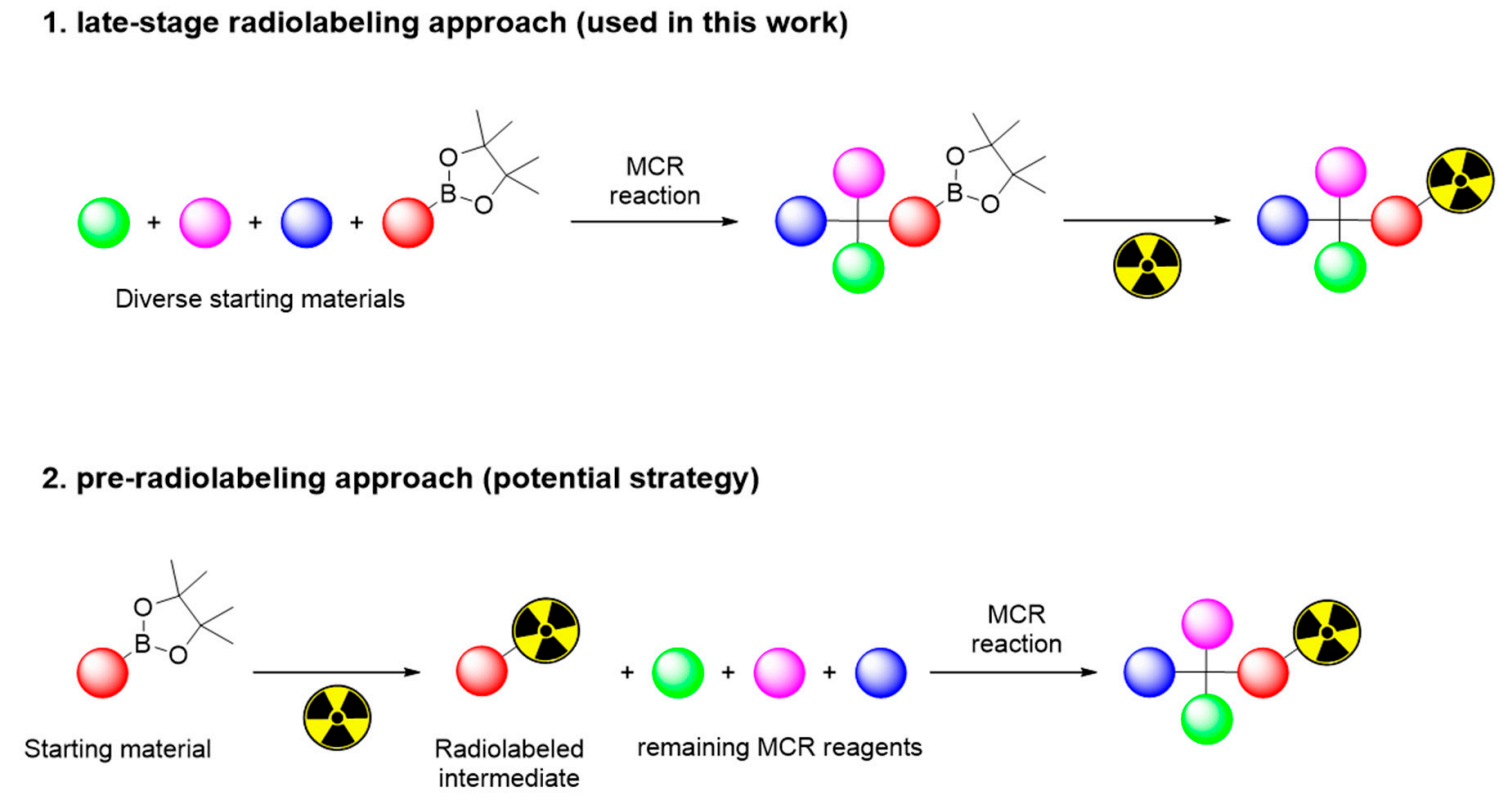
Current and potential multicomponent reaction (MCR) approaches for the generation of scaffold diversity.

Multicomponent reactions (MCRs) can help to solve this problem; they generally involve at least three simple substrates able to react nonsimultaneously, in a one-pot manner, to produce a single final complex structure that can be an organic material, a natural product, or a bioactive molecule. The development of small molecules through convergent MCR has been boosted during the last decade due to the ease of automation and the ability to synthesize numerous small drug-like molecules with several degrees of structural diversity in high yields [4]. It became a powerful tool for the pharmaceutical industry since it provides a quicker, versatile, and more effective way to generate vast libraries of small organic molecules from common intermediate backbones (Figure 1) [5,6]. This allows for a more efficient and time-saving method to achieve molecular diversity, supporting the investigation of how small changes in the overall scaffold may influence functional, biological, and pharmacological activity.

The most established multicomponent assembly processes (e.g., Passerini, Ugi, van Leusen, and Groebke–Blackburn–Bienaymé reactions) rely on isocyanides. However, hundreds of other scaffolds are enabled by MCR [7,8,9,10,11,12]. The usefulness of MCR in the discovery and/or synthesis of drugs and drug-like compounds is clearly supported by the recent advantageous synthesis of Praziquantel, Olanzepine, Ivosidenib, Epelsiban, Retosiban, Lacosamide, and Clopidogrel, just to name a few [13,14,15,16,17,18,19].

Herein, MCR-based synthetic routes have been developed to address the issue of efficient access to PET labeled compounds. Amongst the most used PET isotopes, ^11^C, ^13^N, ^15^O, or ^18^F, we focus here on ^18^F due to its convenient physical half-life (109.7 min). Although several late-stage ^18^F-fluorination strategies have been recently developed (Scheme S1), MCR is underused as a PET tracer assembly strategy [20,21]. The latent automation potential which is based on the usage of the same broadly established aqueous [^18^F]F^−^ pretreatment and [^18^F]KF postprocessing mechanisms similarly to conventional radiochemistry modules, allows for the successful translation to a range of [^18^F]fluoroarenes, including biologically relevant radiotracers [22,23,24,25]. Additionally, the relatively simple synthesis of arylboronates by palladium catalyzed coupling reactions allows the development of arylBPin building blocks that can be further used in MCR [26].

**Scheme 1:**
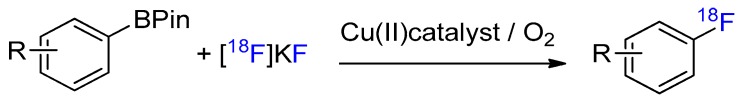
Copper-mediated oxidative ^18^F-fluorination of aryl boronic esters (arylBPin).

Copper-mediated oxidative ^18^F-fluorination of aryl boronic esters (arylBPin) (Scheme 1 and Scheme S2) has been lately revisited and enhanced, showing appropriate tolerance to electron-poor, neutral and rich arenes, and to various functional groups [22,27,28,29].

## 2. Results

**Figure 2:**
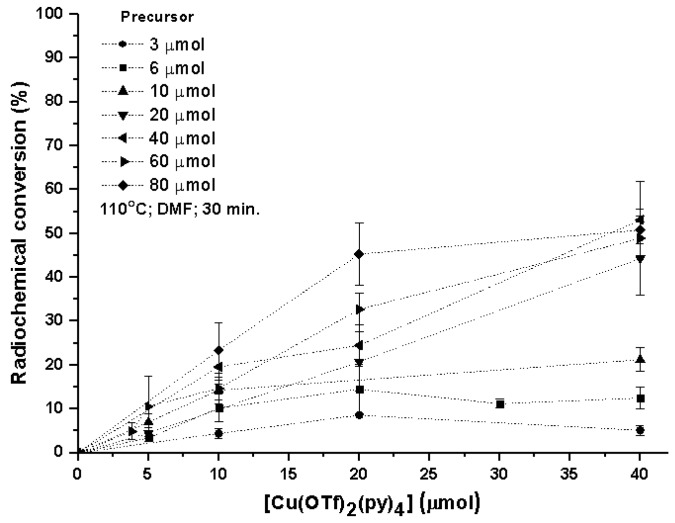
Impact of the Cu catalyst and precursor in the radiochemical conversion (RCC) of 4-[^18^F]fluorobenzaldehyde [^18^F]1 (assessed by radio-TLC).

Testing and implementing this ^18^F-fluorination approach in our lab raised the need for an extensive optimization to identify the variables, bringing more reliable, reproducible, and robust results in a conventional radiochemistry research lab. Therefore, our aim was to investigate the scope and feasibility of this radiolabeling method and specifically use radio-TLC (radio thin-layer chromatography) as a means to identify all radiolabeled products (Table S1) and their radiochemical conversions (RCCs). 4-formylphenylboronic acid pinacol ester was used as a reference substrate for all nonautomated optimization studies. Due to the previously known base sensitivity of Cu-complexes, the standard trapping of aqueous [^18^F]F^−^ to an anion-exchange cartridge was followed by the washout of [(Krypt-222)K^+^][^18^F]F^−^ with an 80% CH_3_CN solution containing cryptand K222 and a very limited amount of K_2_CO_3_ and K_2_C_2_O_4_. [^18^F]fluoride was dried azeotropically, redissolved in DMF (N,N dimethylformamide), transferred to a V-shaped borosilicate reaction vial containing a stirring bar, sealed with a Teflon-lined cap under dry atmospheric air, and placed in an oil bath at 110 °C. Different amounts of [Cu(OTf)_2_(py)_4_] and 4-formylphenylboronic acid pinacol ester precursor were added sequentially to study their influence on the RCC of arylBpin/aryl-^18^F (Figure 2). The duration of the reaction was initially evaluated (at 110 °C) and an ideal time of 30 min was selected for all further reactions. During the first 20 min of reaction it was evident (by following through radio-TLC) a gradual increase of the RCC, reaching a plateau around 30 min, which makes that further prolongation of the reaction till 60 min only brings a very limited increase to the reaction efficiency (<5%).

Predictably, the conversion of [^18^F]fluoride into the wanted radiolabeled products is directly dependent on the amount of catalyst and precursor in the reaction mixture. RCC’s were calculated by radio-TLC where two radiochemical species were identified: the nonreactive [^18^F]F^−^ and the desired 4-[^18^F]fluorobenzaldehyde ([^18^F]1) (Figure S1). We opted to proceed for further studies with a near-optimal proportion of 60 µmol of precursor and 20 µmol of [Cu(OTf)_2_(py)_4_] as an indication and a compromise between precursor to catalyst ratio (3:1). The reaction temperature of Cu-mediated oxidative ^18^F-fluorination in arylBPin has been continuously kept between an optimal 110 °C to 120 °C (see Figure S14 for the effect of temperature in the RCC of [^18^F]1).

The results from our in loco optimization demonstrate that Cu-mediated oxidative ^18^F-fluorination tolerates a range of reaction conditions wider than generally described (temperature and catalyst-to-precursor ratio) [26,27,28]. This has the potential to open future perspectives, turning it into a suitable method for a larger range of molecular backbones which may not handle harsh reaction conditions. Early in the preparation for the optimization studies, it became evident the importance of having the reaction vial flushed with dry air and also of stirring versus gas bubbling or solely heating. Without these procedures, RCC can drop by half.

**Scheme 2:**
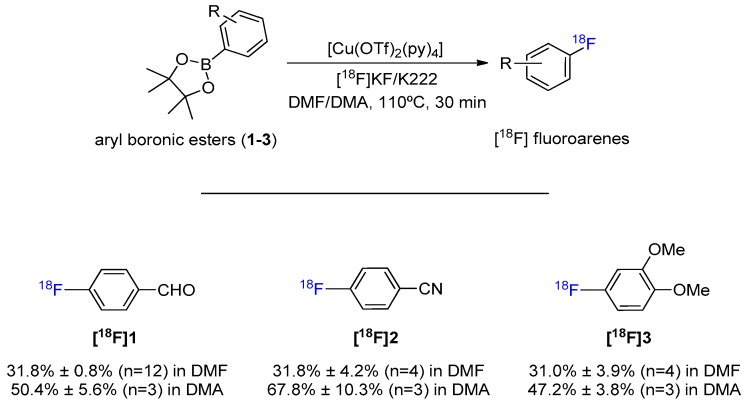
Cu-mediated late-stage ^18^F-fluorination of simple arenes.

The on-site optimized procedure was equally effective for the radiosynthesis of simple electron-deficient ([^18^F]1, [^18^F]2) and electron-rich fluoroarenes ([^18^F]3) (Scheme 2, Figures S1–S3). Cu-mediated oxidative ^18^F-fluorination was subsequently applied to different, more complex MCR scaffolds containing biologically and structurally relevant heterocycles with diverse electrophilic aromatic directing groups. These scaffolds were synthesized to specifically include an aryl boronic acid pinacol ester into the final structure.

**Scheme 3:**
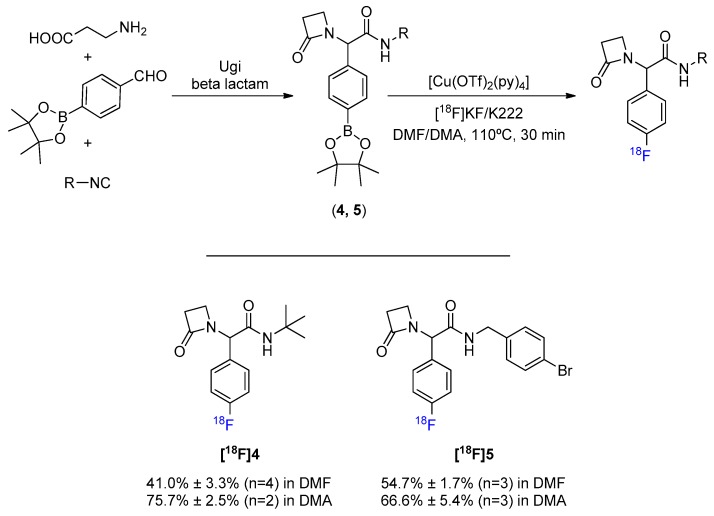
General MCR synthesis and ^18^F-fluorination of β-lactam scaffold.

Despite the emergence of treatment resistance, β-lactams are still among the most successful classes of antibiotics developed so far. This, associated with the fact that the intrinsic strain of four-membered heterocycles turns them more susceptible to hydrolysis, makes the ^18^F-fluorination of a β-lactam scaffold an upright assessment to attest the suitability of the Cu-mediated reaction to more sensitive structures. The synthesis of a β-lactam scaffold was achieved in one step through Ugi’s MCR between the 4-formylphenylboronic acid pinacol ester, β-alanine, and an isocyanide (Scheme 3, Figures S4 and S5). Radiolabeling these products both in DMF and DMA has reiterated the propensity for a better conversion efficacy when using DMA in place of DMF. Consistent results were obtained, indicating that this ^18^F-fluorination methodology may be considered even for scaffolds containing groups with some degree of chemical sensitivity.

**Scheme 4:**
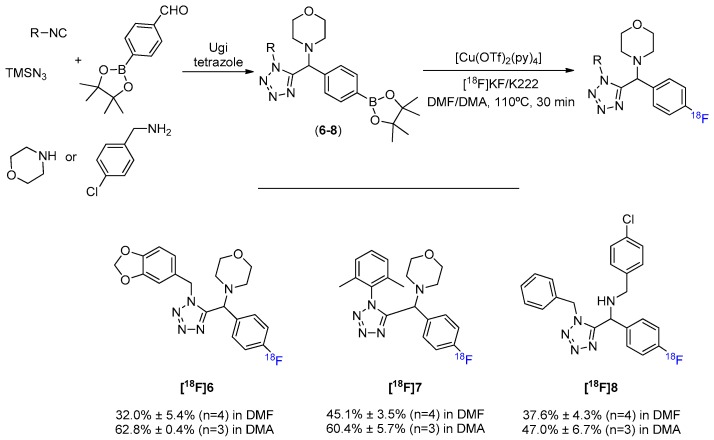
General MCR synthesis and ^18^F-fluorination of tetrazole scaffold.

Next, we synthesized α-amino tetrazoles through the Ugi reaction. As an isosteric substituent of various functional groups (e.g., carboxylate and cis-amide) the tetrazole moiety is often used to confer resistance against the metabolic processes and to increase cell permeability [30]. A tetrazole scaffold containing different complexity in the ring systems involved, which strongly influence the overall electronic and steric properties, was built via Ugi’s four-component condensation and efficiently converted to the [^18^F]fluorinated counterparts (Scheme 4, Figures S6–S8). Interestingly, even the presence of the mobile hydrogen from a secondary amine, which is known to hamper nucleophilic fluorination by causing a decrease in the reactivity of [^18^F]F^−^, does not seem to greatly affect the formation of [^18^F]8. Additionally to the successful conversion of arylBPin derivatives containing a tetrazole function, Cu-mediated ^18^F-fluorination of arylboronate esters also showed to be compatible with the presence of benzodioxoles (antitumoral potential) [31] and morpholines (anti-inflammatory, antimicrobial, anticancer, anti-hyperlipidemic, and analgesic potential) [32].

**Scheme 5:**
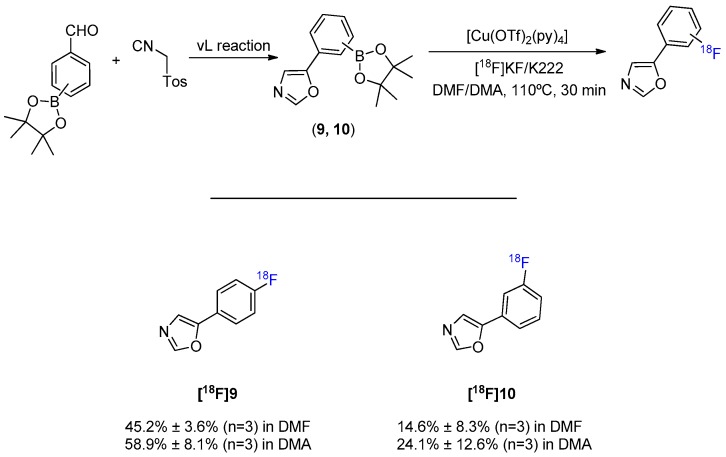
General synthesis and ^18^F-fluorination of oxazole scaffold.

Another class of azoles of great interest as building blocks of pharmaceuticals is the oxazole ring. These can be functional both as synthetic intermediates and as biologically active products (e.g., antibacterial, antifungal, analgesic, anti-inflammatory, hypoglycemic, antiproliferative, antituberculosis, muscle relaxant, and HIV inhibitory activity [33,34,35]). The wide applicability of oxazoles in the synthesis of drugs also justified the evaluation of the compatibility of these aromatic heterocycles with the Cu-mediated oxidative ^18^F-fluorination method. The oxazole scaffold was prepared by the van Leusen (vL) multicomponent reaction between a 4- or 3-formylphenylboronic ester and tosylmethyl isocyanide (Scheme 5). Both para and meta position products were successfully radiolabeled with [^18^F]9 reaching a maximum RCC of 67% and [^18^F]10 a maximum RCC of 38% (Figuress S9 and S10).

**Scheme 6:**
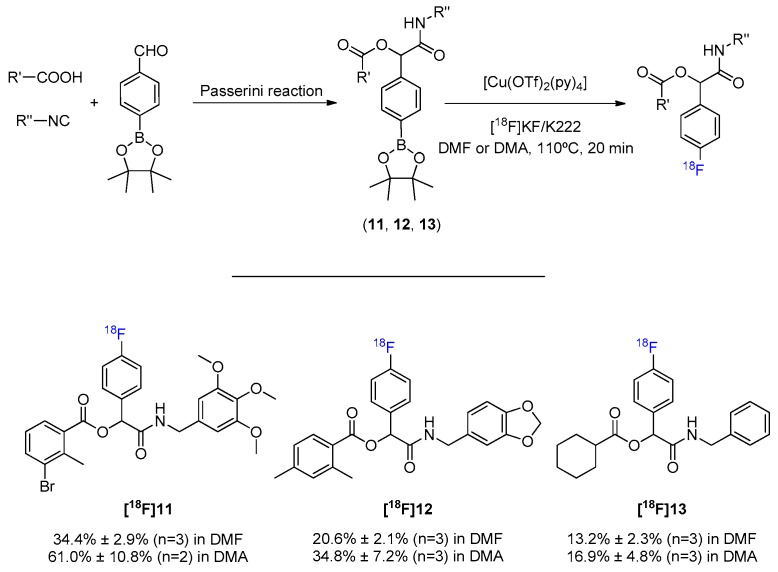
General MCR synthesis and ^18^F-fluorination of α-acyloxy carboxamides.

The Passerini reaction is the oldest isocyanide-based MCR (early 1920s), and has been continuously enhanced being actually a landmark to green chemistry. This versatile three-component reaction of a carboxylic acid, a carbonyl compound, and an isocyanide, gives direct access to α acyloxy carboxamide moieties and has found applications in the field of combinatorial chemistry, natural product and drug synthesis, e.g., inhibitors of HIV-1 protease, antitumoral agents, and fungicides [36]. The latent applicability to the development of small drug-like molecules, allied to the need to foresee how the strong presence of electronegative carboxyl groups and amides, would affect further ^18^F-fluorinations, has motivated the development of arylBPin-containing molecules by Passerini MCR (Scheme 6, Figures S11–S13).

## 3. Discussion

In summary, radiolabeling with fluor-18 was achieved with volumes (800 µL) and activities (up to 2 GBq) compatible with most radiochemistry techniques and automatic modules using oxidative Cu catalysis. ^18^F-Fluorination of aryl boronic ester derivatives was optimized to reach more robust and reproducible reaction conditions capable to offer a wider spectrum of radiolabeling options. These have proved to be successful for a range of temperatures, precursor, and [Cu(OTf)_2_(py)_4_] catalyst amounts, which can be tunable according to the chemical nature of the precursors and to the final aim of the radiotracer. Moreover, we showed the compatibility to several heterocycles commonly used in medicinal chemistry (e.g., β-lactams, tetrazoles, oxazoles, morpholines, and benzodioxoles), which potentiates the ^18^F-fluorination of several small drug-like molecules synthesized through one pot convergent multicomponent reactions. Reproducible RCCs from 15% to 76%, depending on the scaffolds, were achieved, demonstrating the possibility to translate Cu-mediated oxidative ^18^F-fluorination to biologically active molecules synthesized via MCR, allowing latent access to ^18^F-fluorinated drugs. As we firmly confirmed the feasibility of the radiolabeling method, future work will concentrate on the practical aspects of the method including automation and purification.

## 4. Materials and Methods

### 4.1. General Information

Reagents were available from commercial suppliers and used without any purification unless otherwise noted. All isocyanides were made in-house by either performing the Hoffman or Ugi procedure. Other reagents were purchased from Sigma Aldrich, ABCR, Acros, and AK Scientific, and were used without further purification. 

All microwave irradiation reactions were carried out in a Biotage Initiator™ Microwave Synthesizer (Biotage, Uppsala, Sweden) using sealed reaction vessels. The reaction temperature was monitored with an external surface sensor. Electrospray ionization mass spectra (ESI-MS) were recorded on a Waters Investigator Semi-prep 15 SFC-MS instrument (Waters, Milford, MA, USA). HR-MS measurements were recorded on a LTQ-OrbitrapXL at a resolution of 60000 @ m/z 400. Scan range from 150 to 1000 Da. Nuclear magnetic resonance spectra (NMR) were recorded on a Bruker Avance 500 spectrometer (^1^H NMR (500 MHz), ^13^C{^1^H} NMR (126 MHz)) (Bruker, Billerica, MA, USA). Chemical shifts for ^1^H NMR were reported as δ values and coupling constants were reported in hertz (Hz). The following abbreviations were used for spin multiplicity; s = singlet, d = doublet, t = triplet, dd = double doublet, m = multiplet, bs = broad singlet. Chemical shifts for ^13^C NMR were reported in ppm relative to the solvent peak. Thin-layer chromatography was performed on Fluka precoated silica gel plates (0.20 mm thick, particle size 25 μm). Flash chromatography was performed on a Teledyne ISCO Combiflash Rf, using RediSep Rf Normal-phase Silica Flash Columns (Silica Gel 60 Å, 230–400 mesh). 

All of the procedures involving handling of radioactive substances were carried out in a radiochemistry laboratory with the standard required conditions of radiological protection and safety. The use of personal protective equipment and lead shielding, with an appropriate thickness to the manipulated activities, was equally transversal to all experimental radiochemistry procedures. Fluor-18 used in this work was produced by the ^18^O(p,n)^18^F nuclear reaction using an IBA (Louvain-la-Neuve, Belgium) Cyclone 18/9 cyclotron. 

As a final note, all data with standard deviation values presented in this report are the product of at least a duplicate (n ≥ 2) analysis under the same conditions and methodology.

### 4.2. General Procedure for the Synthesis of Compounds

A (Ugi-β-lactam): an aldehyde (1 mmol), β-alanine (1 mmol), and an isocyanide (1 mmol) were added to a sealed microwave reaction vessel. Three milliliters of methanol was added as a solvent and the reaction mixture was left to react for two hours in the microwave at 100 °C. The crude mixture was evaporated and subjected to column chromatography (petroleum ether/ethyl acetate) affording the title compounds.

B (Ugi-Tetrazoles): the corresponding aldehyde (1 mmol), amine (1 mmol), isocyanide (1 mmol), and azidotrimethylsilane (1 mmol) were added to a 5-mL flask along with 3 mL of methanol as a solvent. The reaction mixture was stirred for 24 h at room temperature. The crude mixture was evaporated and subjected to column chromatography (petroleum ether/ethyl acetate) affording the title compounds.

C (van Leusen-Oxazoles): an aldehyde (1 mmol), TosMIC (1 mmol), and K_2_CO_3_ (2 mmol) were added and sealed in a microwave reaction vessel. Three milliliters of methanol was added as a solvent and the reaction mixture was left to react for two hours in the microwave at 100 °C. The crude mixture was evaporated and subjected to column chromatography (petroleum ether/ethyl acetate) affording the title compounds.

D (Passerini): the corresponding aldehyde (1 mmol), acid (1 mmol), and isocyanide (1 mmol) were added to a 5-mL flask along with 3 mL of methanol as a solvent. The reaction mixture was stirred for 24 h at room temperature. The crude mixture was evaporated and subjected to column chromatography (petroleum ether/ethyl acetate) affording the title compounds.

### 4.3. Characterization Data

*4-(4,4,5,5-tetramethyl-1,3,2-dioxaborolan-2-yl)benzaldehyde* (**1**): compound **1** (CAS: 128376-64-7) was purchased from AK Scientific Inc. and was used without further purification.

*4-fluorobenzaldehyde* (**[^19^F]1**): compound **[^19^F]1** (CAS: 459-57-4) was purchased from Sigma Aldrich and was used without further purification.

*4-(4,4,5,5-tetramethyl-1,3,2-dioxaborolan-2-yl)benzonitrile* (**2**): compound **2** (CAS: 171364-82-2) was purchased from FluoroChem and was used without further purification.

*4-fluorobenzonitrile* (**[^19^F]2**): compound **[^19^F]2** (CAS: 1194-02-1) was purchased from AK Scientific Inc. and was used without further purification.

*2-(3,4-dimethoxyphenyl)-4,4,5,5-tetramethyl-1,3,2-dioxaborolane* (**3**): compound **3** (CAS: 365564-10-9) was purchased from AK Scientific Inc. and was used without further purification.

*4-fluoro-1,2-dimethoxybenzene* (**[^19^F]3**): compound **[^19^F]3** (CAS: 398-62-9) was purchased from FluoroChem and was used without further purification.

*N-(tert-butyl)-2-(2-oxoazetidin-1-yl)-2-(4-(4,4,5,5-tetramethyl-1,3,2-dioxaborolan-2-yl)phenyl)acetamide* (**4**): compound 4 was prepared from 4-(4,4,5,5-tetramethyl-1,3,2-dioxaborolan-2-yl)benzaldehyde (232.0 mg), β-alanine (89.1 mg) and tert-Butyl isocyanide (83.1 mg) following the general protocol A maintaining the temperature at 100 °C. Yield: 301.3 mg (78%), yellow oil, ^1^H NMR (500 MHz, CDCl_3_) δ 7.81 (d, *J* = 7.8 Hz, 2H), 7.38 (d, *J* = 7.8 Hz, 2H), 6.67 (s, 1H), 5.54 (s, 1H), 3.70 (td, *J* = 5.6, 2.7 Hz, 1H), 3.11 (td, *J* = 5.6, 2.7 Hz, 1H), 2.93 (ddd, *J* = 14.6, 5.6, 2.7 Hz, 1H), 2.76 (ddd, *J* = 14.6, 5.6, 2.7 Hz, 1H), 1.34 (s, 12H), 1.31 (s, 9H). ^13^C{^1^H} NMR (126 MHz, CDCl_3_) δ 167.8, 167.5, 137.9, 135.1, 127.2, 83.6, 59.0, 51.3, 38.7, 35.9, 28.3, 24.6; HRMS (ESI): *m/z* calcd for C_21_H_32_BN_2_O_4_ [M + H]^+^ 387.2455, found 387.2452.

*N-(tert-butyl)-2-(4-fluorophenyl)-2-(2-oxoazetidin-1-yl)acetamide* (**[^19^F]4**): compound **[^19^F]4** was prepared from 4-fluorobenzaldehyde (124.1 mg), β-alanine (89.1 mg), and tert-Butyl isocyanide (83.1 mg) following the general protocol A maintaining the temperature at 100 °C. Yield: 225.4 mg (81%), white solid, m.p.: 133–135 °C. ^1^H NMR (500 MHz, CDCl_3_) δ 7.38–7.33 (m, 2H), 7.07 (t, *J* = 8.6 Hz, 2H), 6.03 (s, 1H), 5.31 (s, 1H), 3.63 (td, *J* = 5.6, 2.7 Hz, 1H), 3.13 (td, *J* = 5.6, 2.7 Hz, 1H), 3.00 (ddd, *J* = 14.8, 5.6, 2.6 Hz, 1H), 2.85 (ddd, *J* = 14.8, 5.6, 2.6 Hz, 1H), 1.33 (s, 9H). ^13^C{^1^H} NMR (126 MHz, CDCl_3_) δ 167.8, 167.7, 162.7 (d, *J* = 247.9 Hz), 130.9 (d, *J* = 3.3 Hz), 130.0 (d, *J* = 8.3 Hz), 116.0 (d, *J* = 21.7 Hz), 59.3, 51.9, 38.9, 36.3, 28.6; HRMS (ESI): *m/z* calcd for C_15_H_20_FN_2_O_2_ [M + H]^+^ 279.1509, found 279.1502.

*N-(4-bromobenzyl)-2-(2-oxoazetidin-1-yl)-2-(4-(4,4,5,5-tetramethyl-1,3,2-dioxaborolan-2-yl)phenyl)acetamide* (**5**): compound **5** was prepared from 4-(4,4,5,5-tetramethyl-1,3,2-dioxaborolan-2-yl)benzaldehyde (232.0 mg), β-alanine (89.1 mg), and 1-bromo-4-(isocyanomethyl)benzene (196.0 mg) following the general protocol A maintaining the temperature at 100 °C. Yield: 404.3 mg (81%), white solid, m.p.: 97–99 °C. ^1^H NMR (500 MHz, CDCl_3_) δ 7.80 (d, *J* = 7.8 Hz, 2H), 7.66 (t, *J* = 5.7 Hz, 1H), 7.32 (m, 4H), 6.99 (d, *J* = 8.2 Hz, 2H), 5.56 (s, 1H), 4.3–4.18 (m, 2H), 3.63–3.57 (m, 1H), 3.08 (td, *J* = 5.3, 2.4 Hz, 1H), 2.82–2.77 (m, 1H), 2.68–2.63 (m, 1H), 1.34 (s, 12H). ^13^C{^1^H} NMR (126 MHz, CDCl_3_) δ 168.6, 167.7, 137.1, 136.8, 135.2, 131.3, 129.0, 127.2, 120.9, 83.7, 58.8, 42.5, 38.9, 35.8, 24.7; HRMS (ESI): calcd for C_24_H_29_BBrN_2_O_4_ [M + H]^+^ 499.1404, found 499.1398.

*N-(4-bromobenzyl)-2-(4-fluorophenyl)-2-(2-oxoazetidin-1-yl)* (**[^19^F]5**): compound **[^19^F]5** was prepared from 4-fluorobenzaldehyde (124.1 mg), β-alanine (89.1 mg), and 1-bromo-4-(isocyanomethyl)benzene (196.0 mg) following the general protocol A maintaining the temperature at 100 °C. Yield: 293.4 mg (75%), white solid, m.p.: 102–104 °C. ^1^H NMR (500 MHz, CDCl_3_) δ 7.42 (m, 2H), 7.37–7.32 (m, 2H), 7.07 (m, 4H), 6.86 (b, 1H), 5.37 (s, 1H), 4.38 (d, *J* = 5.8 Hz, 2H), 3.58 (td, *J* = 5.6, 2.7 Hz, 1H), 3.16 (td, *J* = 5.6, 2.7 Hz, 1H), 2.96 (ddd, *J* = 14.8, 5.6, 2.7 Hz, 1H), 2.85 (ddd, *J* = 14.8, 5.6, 2.7 Hz, 1H). ^13^C{^1^H} NMR (126 MHz, CDCl_3_) δ 168.7, 168.1, 162.8 (d, *J* = 248.4 Hz), 136.7, 131.8, 130.1 (d, *J* = 3.1 Hz), 130.0 (d, *J* = 8.3 Hz), 129.4, 121.5, 116.2 (d, *J* = 21.7 Hz), 59.4, 43.1, 39.2, 36.2; HRMS (ESI): *m/z* calcd for C_18_H_17_BrFN_2_O_2_ [M + H]^+^ 391.0457, found 391.0455.

*4-((1-(benzo[d][1,3]dioxol-5-ylmethyl)-1H-tetrazol-5-yl)(4-(4,4,5,5-tetramethyl-1,3,2-dioxaborolan-2-yl)phenyl)methyl)morpholine* (**6**): compound **6** was prepared from 4-(4,4,5,5-tetramethyl-1,3,2-dioxaborolan-2-yl)benzaldehyde (232.0 mg), morpholine (87.1 mg), 5-(isocyanomethyl)benzo[d][1,3]dioxole (161.2 mg) and azidotrimethylsilane (115.2 mg) following the general protocol B. Yield: 353.7 mg (70%), orange oil. ^1^H NMR (500 MHz, CDCl_3_) δ 7.76 (d, *J* = 8.0 Hz, 2H), 7.31 (d, *J* = 8.0 Hz, 2H), 6.72 (d, *J* = 8.0 Hz, 1H), 6.58 (dd, *J* = 8.0, 1.1 Hz, 1H), 6.51 (d, *J* = 1.1 Hz, 1H), 5.95 (s, 2H), 5.50 (d, *J* = 15.3 Hz, 1H), 5.29 (d, *J* = 15.3 Hz, 1H), 4.80 (s, 1H), 3.66 (t, *J* = 4.6 Hz, 4H), 2.53–2.44 (m, 2H), 2.36–2.28 (m, 2H), 1.34 (s, 12H). ^13^C{^1^H} NMR (126 MHz, CDCl_3_) δ 153.9, 148.2, 147.9, 136.6, 135.0, 128.3, 126.6, 121.2, 108.3, 107.8, 101.3, 83.9, 66.6, 65.1, 51.2, 50.9, 24.7; HRMS (ESI): *m/z* calcd for C_26_H_33_BN_5_O_5_ [M + H]^+^ 506.2575, found 506.2572.

*4-((1-(benzo[d][1,3]dioxol-5-ylmethyl)-1H-tetrazol-5-yl)(4-fluorophenyl)methyl)morpholine* (**[^19^F]6**): compound **[^19^F]6** was prepared from 4-fluorobenzaldehyde (124.1 mg), morpholine (87.1 mg), 5-(isocyanomethyl)benzo[d][1,3]dioxole (161.2 mg), and azidotrimethylsilane (115.2 mg) following the general protocol B. Yield: 357.6 mg (90%), pale white solid, m.p.: 152–154 °C. ^1^H NMR (500 MHz, CDCl_3_) δ 7.33–7.27 (m, 2H), 7.05–6.95 (m, 2H), 6.75 (d, *J* = 8.0 Hz, 1H), 6.60 (dd, *J* = 8.0, 1.8 Hz, 1H), 6.50 (d, *J* = 1.8 Hz, 1H), 5.97 (dd, *J* = 3.8, 1.4 Hz, 2H), 5.52 (d, *J* = 15.3 Hz, 1H), 5.39 (d, *J* = 15.3 Hz, 1H), 4.74 (s, 1H), 3.81–3.46 (m, 4H), 2.59–2.37 (m, 2H), 2.30 (m, 2H). ^13^C{^1^H} NMR (126 MHz, CDCl_3_) δ 162.6 (d, *J* = 248.5 Hz), 153.9, 148.1 (d, *J* = 33.5 Hz), 130.9 (d, *J* = 8.2 Hz), 129.4, 126.7, 121.1, 115.5 (d, *J* = 21.6 Hz), 108.3, 107.7, 101.4, 66.6, 64.1, 50.9; HRMS (ESI): calcd for C_20_H_21_FN_5_O_3_ [M + H]^+^ 398.1628, found 398.1621.

*4-((1-(2,6-dimethylphenyl)-1H-tetrazol-5-yl)(4-(4,4,5,5-tetramethyl-1,3,2-dioxaborolan-2-yl)phenyl)methyl)morpholine* (**7**):

Compound **7** was prepared from 4-(4,4,5,5-tetramethyl-1,3,2-dioxaborolan-2-yl)benzaldehyde (232.0 mg), morpholine (87.1 mg), 2-isocyano-1,3-dimethylbenzene (131.2 mg), and azidotrimethylsilane (115.2 mg) following the general protocol B. Yield: 423.1 mg (89%), white solid, m.p.: 155–157 °C. ^1^H NMR (500 MHz, CDCl_3_) δ 7.71 (d, *J* = 7.6 Hz, 2H), 7.39 (t, *J* = 7.6 Hz, 1H), 7.29 (d, *J* = 7.7 Hz, 1H), 7.20 (d, *J* = 7.6 Hz, 2H), 7.08 (d, *J* = 7.6 Hz, 1H), 4.28 (s, 1H), 3.70 (m, 4H), 2.61–2.35 (m, 4H), 2.05 (s, 3H), 1.33 (d, *J* = 4.2 Hz, 12H), 1.11 (s, 3H). ^13^C{^1^H} NMR (126 MHz, CDCl_3_) δ 155.0, 136.9, 136.4, 134.7, 134.5, 131.0, 130.8, 128.6, 128.5, 128.4, 83.6, 66.3, 65.3, 51.5, 24.6, 17.2, 16.2; HRMS (ESI): *m/z* calcd for C_26_H_35_BN_5_O_3_ [M + H]^+^ 476.2833, found 476.2831.

*4-((1-(2,6-dimethylphenyl)-1H-tetrazol-5-yl)(4-fluorophenyl)methyl)morpholine* (**[^19^F]7**): compound **[^19^F]7** was prepared from 4-fluorobenzaldehyde (124.1 mg), morpholine (87.1 mg), 2-isocyano-1,3-dimethylbenzene (131.2 mg), and azidotrimethylsilane (115.2 mg) following the general protocol B. Yield: 323.3 mg (88%), white solid, m.p.: 123–125 °C. ^1^H NMR (500 MHz, CDCl_3_) δ 7.41 (t, *J* = 7.6 Hz, 1H), 7.30 (d, *J* = 7.6 Hz, 1H), 7.26–7.17 (m, 2H), 7.12 (d, *J* = 7.6 Hz, 1H), 6.96 (t, *J* = 8.6 Hz, 2H), 4.30 (s, 1H), 3.74–3.67 (m, 4H), 2.72–2.33 (m, 4H), 2.05 (s, 3H), 1.20 (s, 3H). ^13^C{^1^H} NMR (126 MHz, CDCl_3_) δ 162.4 (d, *J* = 248.9 Hz), 155.0, 136.1, 134.6, 131.0, 129.9 (d, *J* = 3.2 Hz), 128.6 (d, *J* = 2.7 Hz), 115.3 (d, *J* = 21.5 Hz), 66.3, 64.4, 51.3, 17.2, 16.2; HRMS (ESI): *m/z* calcd for C_20_H_23_FN_5_O [M + H]^+^ 368.1887, found 368.1883.

*1-(1-benzyl-1H-tetrazol-5-yl)-N-(4-chlorobenzyl)-1-(4-(4,4,5,5-tetramethyl-1,3,2-dioxaborolan-2-yl)phenyl)methanamine* (**8**): compound **8** was prepared from 4-(4,4,5,5-tetramethyl-1,3,2-dioxaborolan-2-yl)benzaldehyde (232.0 mg), (4-chlorophenyl)methanamine (141.6 mg), (isocyanomethyl)benzene (117.2 mg), and azidotrimethylsilane (115.2 mg) following the general protocol B. Yield: 366.2 mg (71%), pale yellow solid, m.p.: 111–113 °C. ^1^H NMR (500 MHz, CDCl_3_) δ 7.77 (d, *J* = 8.0 Hz, 2H), 7.33–7.27 (m, 1H), 7.26–7.21 (m, 4H), 7.16 (d, *J* = 8.0 Hz, 2H), 7.08 (d, *J* = 8.3 Hz, 2H), 6.92 (d, *J* = 7.2 Hz, 2H), 5.38 (d, *J* = 15.4 Hz, 1H), 5.12 (d, *J* = 15.4 Hz, 1H), 4.91 (s, 1H), 3.66–3.55 (m, 2H), 1.34 (s, 12H). ^13^C{^1^H} NMR (126 MHz, CDCl_3_) δ 155.4, 139.9, 137.2, 135.6, 133.0, 129.6, 129.1, 128.8, 128.6, 127.4, 126.9, 84.1, 55.8, 51.0, 50.2, 24.9; HRMS (ESI): *m/z* calcd for C_28_H_32_BClN_5_O_2_ [M + H]^+^ 516.2338, found 516.2332.

*1-(1-benzyl-1H-tetrazol-5-yl)-N-(4-chlorobenzyl)-1-(4-fluorophenyl)methanamine* (**[^19^F]8**): compound **[^19^F]8** was prepared from 4-fluorobenzaldehyde (124.1 mg), (4-chlorophenyl)methanamine (141.6 mg), (isocyanomethyl)benzene (117.2 mg), and azidotrimethylsilane (115.2 mg) following the general protocol B. Yield: 322.2 mg (79%), pale yellow solid, m.p.: 73–75 °C. ^1^H NMR (500 MHz, CDCl_3_) δ 7.30 (m, 1H), 7.25 (m, 4H), 7.14–7.07 (m, 4H), 7.02–6.96 (m, 2H), 6.90 (d, *J* = 7.3 Hz, 2H), 5.40 (d, *J* = 15.5 Hz, 1H), 5.25 (d, *J* = 15.5 Hz, 1H), 4.89 (s, 1H), 3.60 (s, 2H). ^13^C{^1^H} NMR (126 MHz, CDCl_3_) δ 162.7 (d, *J* = 248.4 Hz), 155.4, 137.0, 133.1 (d, *J* = 24.2 Hz), 132.9, 129.6, 129.4 (d, *J* = 8.3 Hz), 129.2, 128.9, 128.7, 127.3, 116.1 (d, *J* = 21.8 Hz), 55.0, 51.0, 50.3; HRMS (ESI): *m/z* calcd for C_22_H_20_ClFN_5_ [M + H]^+^ 408.1391, found 408.1386.

*5-(4-(4,4,5,5-tetramethyl-1,3,2-dioxaborolan-2-yl)phenyl)oxazole* (**9**): compound **9** was prepared from 4-(4,4,5,5-tetramethyl-1,3,2-dioxaborolan-2-yl)benzaldehyde (232.0 mg), p-Toluenesulfonylmethyl isocyanide (195.2 mg) and K_2_CO_3_ (276.4 mg) following the general protocol C maintaining the temperature at 100 °C. Yield: 252.1 mg (93%), white solid, m.p.: 130–132 °C. ^1^H NMR (500 MHz, CDCl_3_) δ 7.93 (s, 1H), 7.86 (d, *J* = 8.1 Hz, 2H), 7.66 (d, *J* = 8.1 Hz, 2H), 7.41 (s, 1H), 1.36 (s, 12H). ^13^C{^1^H} NMR (126 MHz, CDCl_3_) δ 151.5, 150.7, 135.3, 130.1, 123.5, 122.3, 84.0, 24.9; HRMS (ESI): *m/z* calcd for C_15_H_19_BNO_3_ [M + H]^+^ 272.1458, found 272.1453.

*5-(4-fluorophenyl)oxazole* (**[^19^F]9**): compound **[^19^F]9** was prepared from 4-fluorobenzaldehyde (124.1 mg), p-Toluenesulfonylmethyl isocyanide (195.2 mg), and K_2_CO_3_ (276.4 mg) following the general protocol C maintaining the temperature at 100 °C. Yield: 145.2 mg (89%), red oil. ^1^H NMR (500 MHz, CDCl_3_) δ 7.90 (s, 1H), 7.59–7.48 (m, 2H), 7.28 (s, 1H), 7.14–6.97 (t, *J* = 8.7 Hz, 2H). ^13^C{^1^H} NMR (126 MHz, CDCl_3_) δ 162.2 (d, *J* = 248.8 Hz), 150.2, 150.0, 125.7 (d, *J* = 8.2 Hz), 123.6 (d, *J* = 3.4 Hz), 120.7, 115.5 (d, *J* = 22.2 Hz); HRMS (ESI): *m/z* calcd for C_9_H_6_FNO [M + H]^+^ 164.0512, found 164.0508.

*5-(3-(4,4,5,5-tetramethyl-1,3,2-dioxaborolan-2-yl)phenyl)oxazole* (**10**): compound **10** was prepared from 3-(4,4,5,5-tetramethyl-1,3,2-dioxaborolan-2-yl)benzaldehyde (232.0 mg), p-Toluenesulfonylmethyl isocyanide (195.2 mg), and K_2_CO_3_ (276.4 mg) following the general protocol C maintaining the temperature at 100 °C. Yield: 216.8 mg (80%), red oil. ^1^H NMR (500 MHz, CDCl_3_) δ 8.09 (s, 1H), 7.91 (s, 1H), 7.79 (d, *J* = 7.6 Hz, 1H), 7.72 (d, *J* = 7.6 Hz, 1H), 7.42 (t, *J* = 7.6 Hz, 1H), 7.38 (s, 1H), 1.35 (s, 12H). ^13^C{^1^H} NMR (126 MHz, CDCl_3_) δ 151.4, 150.4, 134.8, 130.5, 128.2, 127.0, 126.9, 121.3, 83.9, 24.7; HRMS (ESI): *m/z* calcd for C_15_H_19_BNO_3_ [M + H]^+^ 272.1458, found 272.1453.

*5-(3-fluorophenyl)oxazole* (**[^19^F]10**): compound **[^19^F]10** was prepared from 3-fluorobenzaldehyde (232.0 mg), p-Toluenesulfonylmethyl isocyanide (195.2 mg), and K_2_CO_3_ (276.4 mg) following the general protocol C maintaining the temperature at 100 °C. Yield: 122.4 mg (75%), red oil. ^1^H NMR (500 MHz, CDCl_3_) δ 7.93 (s, 1H), 7.44 (dt, *J* = 7.8, 1.2 Hz, 1H), 7.42–7.38 (m, 2H), 7.38–7.34 (m, 1H), 7.07–7.02 (m, 1H). ^13^C{^1^H} NMR (126 MHz, CDCl_3_) δ 163.1 (d, *J* = 246.4 Hz), 150.9, 130.7 (d, *J* = 8.5 Hz), 129.6 (d, *J* = 8.5 Hz), 122.3, 120.1 (d, *J* = 2.8 Hz), 115.6 (d, *J* = 21.4 Hz), 111.4 (d, *J* = 23.8 Hz); HRMS (ESI): *m/z* calcd for C_9_H_7_FNO [M + H]^+^ 164.0512, found 164.0507.

*2-oxo-1-(4-(4,4,5,5-tetramethyl-1,3,2-dioxaborolan-2-yl)phenyl)-2-((3,4,5-trimethoxybenzyl)amino)ethyl 3-bromo-2-methylbenzoate* (**11**): compound **11** was prepared from 4-(4,4,5,5-tetramethyl-1,3,2-dioxaborolan-2-yl)benzaldehyde (232.0 mg), 3-bromo-2-methylbenzoic acid (215.0 mg), and 5-(isocyanomethyl)-1,2,3-trimethoxybenzene (207.2 mg) following the general protocol D. Yield: 536.6 mg (82%), white solid, m.p.: 137–139 °C. ^1^H NMR (500 MHz, CDCl_3_) δ 7.85 (d, *J* = 8.1 Hz, 2H), 7.79 (dd, *J* = 8.0, 1.0 Hz, 1H), 7.72 (dd, *J* = 8.0, 1.1 Hz, 1H), 7.56 (d, *J* = 8.1 Hz, 2H), 7.11 (t, *J* = 8.0 Hz, 1H), 6.35 (s, 2H), 6.26 (s, 1H), 4.47–4.37 (m, 2H), 3.80 (s, 3H), 3.73 (s, 6H), 2.60 (s, 3H), 1.34 (s, 12H). ^13^C{^1^H} NMR (126 MHz, CDCl_3_) δ 168.1, 165.9, 153.4, 139.1, 137.8, 137.2, 136.5, 135.4, 133.5, 131.6, 129.3, 127.2, 126.9, 126.6, 104.1, 84.1, 60.8, 56.0, 43.4, 24.9, 20.7; HRMS (ESI): *m/z* calcd for C_32_H_38_BBrNO_8_ [M + H]^+^ 654.1874, found 654.1869.

*1-(4-fluorophenyl)-2-oxo-2-((3,4,5-trimethoxybenzyl)amino)ethyl 3-bromo-2-methylbenzoate* (**[^19^F]11**): compound **[^19^F]11** was prepared from 4-fluorobenzaldehyde (124.1 mg), 3-bromo-2-methylbenzoic acid (215.0 mg), and 5-(isocyanomethyl)-1,2,3-trimethoxybenzene (207.2 mg) following the general protocol D. Yield: 459.0 mg (84%), white solid, m.p.: 120–122 °C. ^1^H NMR (500 MHz, CDCl_3_) δ 7.78 (d, *J* = 7.8 Hz, 1H), 7.70 (d, *J* = 7.8 Hz, 1H), 7.58–7.52 (m, 2H), 7.07 (m, 3H), 6.83 (t, *J* = 6.0 Hz, 1H), 6.36 (s, 2H), 6.21 (s, 1H), 4.43 (dd, *J* = 15.1, 6.0 Hz, 1H), 4.32 (dd, *J* = 15.1, 6.0 Hz, 1H), 3.79 (s, 3H), 3.73 (s, 6H), 2.59 (s, 3H). ^13^C{^1^H} NMR (126 MHz, CDCl_3_) δ 168.3, 165.9, 163.1 (d, *J* = 248.7 Hz), 153.3, 139.0, 136.4, 133.5, 131.4, 131.0 (d, *J* = 3.3 Hz), 129.4 (d, *J* = 8.4 Hz), 129.2, 127.2, 126.9, 115.8 (d, *J* = 21.8 Hz), 104.0, 75.8, 60.7, 55.9, 43.3, 20.5; HRMS (ESI): *m/z* calcd for C_26_H_26_BrFNO_6_ [M + H]^+^ 546.0928, found 546.0923.

*2-((benzo[d][1,3]dioxol-5-ylmethyl)amino)-2-oxo-1-(4-(4,4,5,5-tetramethyl-1,3,2-dioxaborolan-2-yl)phenyl)ethyl 2,4-dimethylbenzoate* (**12**): compound **12** was prepared from 4-(4,4,5,5-tetramethyl-1,3,2-dioxaborolan-2-yl)benzaldehyde (232.0 mg), 2,4-dimethylbenzoic acid (150.2 mg), and 5-(isocyanomethyl)benzo[d][1,3]dioxole (161.2 mg) following the general protocol D. Yield: 472.7 mg (87%), white solid. M.p.: 134–136 °C. ^1^H NMR (500 MHz, CDCl_3_) δ 7.85 (m, 3H), 7.53 (d, *J* = 8.0 Hz, 2H), 7.06 (m, 2H), 6.75–6.65 (m, 3H), 6.36 (t, *J* = 5.7 Hz, 1H), 6.31 (s, 1H), 5.95–5.92 (m, 2H), 4.38 (d, *J* = 5.9 Hz, 2H), 2.54 (s, 3H), 2.35 (s, 3H), 1.34 (s, 12H). ^13^C{^1^H} NMR (126 MHz, CDCl_3_) δ 168.3, 165.5, 148.0, 147.1, 143.4, 141.1, 138.5, 135.3, 132.8, 131.6, 130.9, 126.7, 126.6, 125.5, 121.0, 108.4, 108.3, 101.1, 84.0, 75.8, 43.3, 24.9, 21.8, 21.5; HRMS (ESI): *m/z* calcd for C_31_H_35_BNO_7_ [M + H]^+^ 544.2507, found 544.2502.

*2-((benzo[d][1,3]dioxol-5-ylmethyl)amino)-1-(4-fluorophenyl)-2-oxoethyl 2,4-dimethylbenzoate* (**[^19^F]12**): compound **[^19^F]12** was prepared from 4-fluorobenzaldehyde (124.1 mg), 2,4-dimethylbenzoic acid (150.2 mg), and 5-(isocyanomethyl)benzo[d][1,3]dioxole (161.2 mg) following the general protocol D. Yield: 374.5 mg (86%), white solid, m.p.: 139–141 °C. ^1^H NMR (500 MHz, CDCl_3_) δ 7.84 (d, *J* = 7.9 Hz, 1H), 7.54–7.47 (m, 2H), 7.10–7.05 (m, 4H), 6.76–6.67 (m, 3H), 6.45 (m, 1H), 6.29 (s, 1H), 5.95–5.93 (m, 2H), 4.40 (m, *J* = 5.9 Hz, 2H), 2.54 (s, 3H), 2.36 (s, 3H). ^13^C{^1^H} NMR (126 MHz, CDCl_3_) δ 168.4, 165.5, 163.0 (d, *J* = 248.0 Hz), 148.0, 147.1, 143.6, 141.2, 132.8, 131.7 (d, *J* = 3.2 Hz), 131.5, 130.8, 129.3 (d, *J* = 8.4 Hz), 126.7, 125.3, 121.0, 115.8 (d, *J* = 21.7 Hz), 108.4, 108.3, 101.1, 75.0, 43.3, 21.8, 21.5; HRMS (ESI): *m/z* calcd for C_25_H_23_FNO_5_: [M + H]^+^ 436.1560, found 436.1558.

*2-(benzylamino)-2-oxo-1-(4-(4,4,5,5-tetramethyl-1,3,2-dioxaborolan-2-yl)phenyl)ethyl cyclohexanecarboxylate* (**13**): compound **13** was prepared from 4-(4,4,5,5-tetramethyl-1,3,2-dioxaborolan-2-yl)benzaldehyde (232.0 mg), cyclohexanecarboxylic acid (128.2 mg), and (isocyanomethyl)benzene (117.2 mg) following the general protocol D. Yield: 324.6 mg (68%), transparent oil. ^1^H NMR (500 MHz, CDCl_3_) δ 7.81 (d, *J* = 8.0 Hz, 2H), 7.44 (d, *J* = 8.0 Hz, 2H), 7.36–7.27 (m, 3H), 7.22 (d, *J* = 6.9 Hz, 2H), 6.32 (t, *J* = 5.6 Hz, 1H), 6.13 (s, 1H), 4.47 (d, *J* = 5.8 Hz, 2H), 2.44–2.38 (m, 1H), 1.96–1.85 (m, 2H), 1.74 (m, 2H), 1.63 (m,1H), 1.43 (m, 3H), 1.34 (s, 12H), 1.31–1.16 (m, 4H). ^13^C{^1^H} NMR (126 MHz, CDCl_3_) δ 174.1, 168.2, 138.5, 137.7, 135.0, 134.9, 128.5, 127.4, 127.3, 126.3, 125.7, 83.7, 75.0, 72.8, 52.7, 43.1, 42.6, 28.7, 28.6, 25.4, 25.1, 25.0, 24.7; HRMS (ESI): *m/z* calcd for C_28_H_37_BNO_5_ [M + H]^+^ 478.2765, found 478.2762.

*2-(benzylamino)-1-(4-fluorophenyl)-2-oxoethyl cyclohexanecarboxylate* (**[^19^F]13**): compound **[^19^F]13** was prepared from 4-fluorobenzaldehyde (124.1 mg), cyclohexanecarboxylic acid (128.2 mg), and (isocyanomethyl)benzene (117.2 mg) following the general protocol D. Yield: 255.0 mg (69%), pale yellow oil. ^1^H NMR (500 MHz, CDCl_3_) δ 7.43–7.37 (m, 2H), 7.31–7.21 (m, 3H), 7.19–7.13 (m, 2H), 7.03–6.97 (m, 2H), 6.84 (t, *J* = 5.7 Hz, 1H), 6.06 (s, 1H), 4.46–4.31 (m, 2H), 2.41–2.35 (m, 1H), 1.91–1.85 (m, 2H), 1.77–1.66 (m, 2H), 1.61 (dd, *J* = 11.1, 3.8 Hz, 1H), 1.49–1.33 (m, 2H), 1.32–1.12 (m, 3H). ^13^C{^1^H} NMR (126 MHz, CDCl_3_) δ 180.3, 174.2, 168.5, 162.8 (d, *J* = 247.8 Hz), 137.6, 131.5 (d, *J* = 3.2 Hz), 129.0 (d, *J* = 8.3 Hz), 128.5, 127.4, 127.3, 115.46 (d, *J* = 21.5 Hz), 74.3, 43.1, 42.6, 28.7, 28.6, 25.4, 25.1; HRMS (ESI): *m/z* calcd for C_22_H_25_FNO_3_ [M + H]^+^ 370.1818, found 370.1812.

### 4.4. General Procedure for the Radiolabeling Chemistry

General procedure for the optimization of ^18^F-labeling of arylBPin derivatives: ^18^F-fluorination of boronic acid pinacol esters was first optimized, in a nonautomated manner, using 4-formylphenylboronic acid pinacol ester (**1**) in relation to the ideal amount of [Cu(OTf)_2_(py)_4_] catalyst and precursor to be used, as well as the reaction solvent and temperature. Each intended amount of **1** (3 to 80 µmol) and [Cu(OTf)_2_(py)_4_] (4 to 40 µmol) was dissolved in 150 µL of anhydrous DMF (or DMA) and the resulting solutions loaded into 1.0 mL syringes. Aqueous [^18^F]fluoride from the cyclotron (≤2 GBq) was loaded onto an anion exchange cartridge (Chromafix PS-HCO_3_) and then washed out to a reaction V-vial (containing a stirring bar) with 1 mL of an 80% CH_3_CN solution of 3.15 mg K222, 0.05 mg K_2_CO_3_, and 0.5 mg K_2_C_2_O_4_. The mixture was submitted to evaporation by azeotropic distillation. Initially 1 mL anhydrous CH_3_CN was added to the [(Krypt-222)K^+^][^18^F]F^−^ solution recovered from the anion exchange cartridge and left to dry at 110 °C with constant stirring and under a flow of argon (dried with P_2_O_5_/ascarite). After total drying, 0.5 mL of anhydrous CH_3_CN was added and left to dry to the completion again. This step was repeated four more times. The reaction V-vial containing dried [(Krypt-222)K^+^][^18^F]F^−^ was then purged with 10 mL of dried atmospheric air (passed through a P_2_O_5_ cartridge) and dissolved in 0.5 mL of anhydrous DMF (or DMA). [(Krypt-222)K^+^][^18^F]F^−^ was dissolved in the organic solvent was left (under stirring, bubbling with dry air or without a mixing system) at the reaction temperature (22 °C to 170 °C). Subsequently, 150 µL of the [Cu(OTf)_2_(py)_4_] solution and 150 µL of **1** were added to the reaction vial (total volume of 800 µL; see Figure 2 for catalyst-to-precursor ratio impact). The reaction was followed up to 40 min. Product formation was characterized by comparing the retention factors (Rf) of the crude reaction mixture (in a TLC-SG developed with a 2:1 hexane:ethyl acetate mobile phase) alongside the authentic, nonradioactive, but UV visible (254 nm), reference sample (4-fluorobenzaldehyde) spiked after development with a radioactive spot. Radiochemical yields of the conversion to the ^18^F-fluorinated species (RCC) were also assessed through this chromatographic technique. Radio-TLC’s were scanned using a Perkin Elmer Packard Cyclone^®^ storage phosphor system and the acquired data analyzed with the OptiQuant 04.00 software.

Nonautomated procedure for the ^18^F-labeling of MCR arylBPin derivatives: work-up of aqueous [^18^F]fluoride to [(Krypt-222)K^+^][^18^F]F^−^ was followed in accordance with the general procedure for the optimization of ^18^F-labeling of arylBPin derivatives. The near-optimal method chosen for the radiolabeling of the MCR scaffolds started with the preparation of a V-vial at 110 °C containing a magnetic stirrer and [(Krypt 222)K^+^][^18^F]F^−^ (≤2 GBq) in DMF or DMA (ca. 500 μL). This vial was sealed and purged with 10 mL of dried atmospheric air (through a P_2_O_5_ cartridge). Subsequently, 150 μL of [Cu(OTf)_2_(py)_4_] (0.02 mmol in anhydrous DMF or DMA) and 150 μL arylBPin precursor (0.06 mmol in anhydrous DMF or DMA) were added and allowed to stir for 30 min in an oil bath. The reaction was quenched by addition of water (500 μL) and an aliquot was taken for analysis by radio thin-layer chromatography silica gel, developed with hexane:ethyl acetate, to calculate the RCC and identify the product (UV 254 nm).

## Supplementary Materials

The following are available online. Figures S1–S13: Examples of the radio-TLCs; Table S1: TLC-SG retention factor (Rf) profiles for the tested compounds; Figure S14: Effect of temperature in RCC of **[^18^F]1**; Scheme S1: Recent late-stage strategies for the ^18^F-fluorination of (hetero)arenes; Scheme S2: Proposed mechanism for copper-mediated oxidative ^18^F-fluorination of aryl boronic esters; Spectra of compounds.

## References

[1] Matthews P.M., Rabiner E.A., Passchier J., Gunn R.N. (2012). Positron emission tomography molecular imaging for drug development. Brit. J. Clin. Pharmacol..

[2] Fernandes E., Barbosa Z., Clemente G., Alves F., Abrunhosa A.J. (2012). Positron emitting tracers in pre-clinical drug development. Curr. Rad..

[3] Patel S., Schmidt K., Hesterman J., Hoppin J. (2017). Advancing Drug Discovery and Development Using Molecular Imaging (ADDMI): An Interest Group of the World Molecular Imaging Society and an Inaugural Session on Positron Emission Tomography (PET). Mol. Imaging Biol..

[4] Kalinski C., Umkehrer M., Weber L., Kolb J., Burdack C., Ross G. (2010). On the industrial applications of MCRs: Molecular diversity in drug discovery and generic drug synthesis. Mol. Divers..

[5] Slobbe P., Ruijter E., Orru R.V.A. (2012). Recent applications of multicomponent reactions in medicinal chemistry. MedChemComm.

[6] Zarganes-Tzitzikas T., Domling A. (2014). Modern multicomponent reactions for better drug syntheses. Org. Chem. Front..

[7] Ruijter E., Scheffelaar R., Orru R.V.A. (2011). Multicomponent Reaction Design in the Quest for Molecular Complexity and Diversity. Angew. Chem. Int. Ed..

[8] Dömling A., Wang W., Wang K. (2012). Chemistry and Biology Of Multicomponent Reactions. Chem. Rev..

[9] De Graaff C., Ruijter E., Orru R.V.A. (2012). Recent developments in asymmetric multicomponent reactions. Chem. Soc. Rev..

[10] Dömling A., AlQahtani A.D., Zhu J., Wang Q., Wang M. (2014). Multicomponent Reactions in Organic Synthesis. Multicomponent Reactions in Organic Synthesis.

[11] Alegre-Requena J.V., Marqués-López E., Herrera R.P., Herrera R.P., Marqués-López E. (2015). Introduction: Multicomponent Startegies. Multicomponent Reactions: Concepts and Applications for Design and Synthesis.

[12] Zhu Q., Yuan Q., Chen M., Guo M., Huang H. (2017). Multicomponent Reactions with Cyclic Tertiary Amines Enabled by Facile C−N Bond Cleavage. Angew. Chem. Int. Ed..

[13] Wang K., Nguyen K., Huang Y., Dömling A. (2009). Cyanoacetamide Multicomponent Reaction (I): Parallel Synthesis Of Cyanoacetamides. J. Comb. Chem..

[14] Cao H., Liu H., Dömling A. (2010). Efficient Multicomponent Reaction Synthesis of the Schistosomiasis Drug Praziquantel. Chem. Eur. J..

[15] Aillaud I., Haurena C., Gall E.L., Martens T., Ricci G. (2010). 2-Chlorophenyl Zinc Bromide: A Convenient Nucleophile for the Mannich-Related Multicomponent Synthesis of Clopidogrel and Ticlopidine. Molecules.

[16] Liu H., William S., Herdtweck E., Botros S., Dömling A. (2012). MCR Synthesis of Praziquantel Derivatives. Chem. Biol. Drug Des..

[17] Khoury K., Sinha M.K., Nagashima T., Herdtweck E., Dömling A. (2012). Efficient assembly of iminodicarboxamides by a truly four component reaction. Angew. Chem. Int. Ed..

[18] Neochoritis C.G., Stotani S., Mishra B., Dömling A. (2015). Efficient Isocyanide-less Isocyanide-Based Multicomponent Reactions. Org. Let..

[19] Wehlan H., Oehme J., Schäfer A., Rossen K. (2015). Development of Scalable Conditions for the Ugi Reaction—Application to the Synthesis of (R)-Lacosamide. Org. Process Res. Dev..

[20] Li L., Hopkinson M.N., Yona R.L., Bejot R., Gee A.D., Gouverneur V. (2011). Convergent ^18^F radiosynthesis: A new dimension for radiolabelling. Chem. Sci..

[21] Qu W., Zha Z., Ploessl K., Lieberman B.P., Zhu L., Wise D.R., Thompson C.B., Kung H.F. (2011). Synthesis of Optically Pure 4-Fluoro-Glutamines as Potential Metabolic Imaging Agents for Tumors. J. Am. Chem. Soc..

[22] Preshlock S., Calderwood S., Verhoog S., Tredwell M., Huiban M., Hienzsch A., Gruber S., Wilson T.C., Taylor N.J., Cailly T. (2016). Enhanced copper-mediated ^18^F-fluorination of aryl boronic esters provides eight radiotracers for PET applications. Chem. Commun..

[23] Schäfer D., Weiß P., Ermert J., Castillo Meleán J., Zarrad F., Neumaier B. (2016). Preparation of No-Carrier-Added 6-[^18^F]Fluoro-l-tryptophan via Cu-Mediated Radiofluorination. Eur. J. Org. Chem..

[24] Giglio B.C., Fei H., Wang M., Wang H., He L., Feng H., Wu Z., Lu H., Li Z. (2017). Synthesis of 5-[(18)F]Fluoro-α-methyl Tryptophan: New Trp Based PET Agents. Theranostics.

[25] Taylor N.J., Emer E., Preshlock S., Schedler M., Tredwell M., Verhoog S., Mercier J., Genicot C., Gouverneur V. (2017). Derisking the Cu-Mediated ^18^F-Fluorination of Heterocyclic Positron Emission Tomography Radioligands. J. Am. Chem. Soc..

[26] Lu J., Guan Z.-Z., Gao J.-W., Zhang Z.-H. (2011). An improved procedure for the synthesis of arylboronates by palladium-catalyzed coupling reaction of aryl halides and bis(pinacolato)diboron in polyethylene glycol. Appl. Org. Chem..

[27] Tredwell M., Preshlock S.M., Taylor N.J., Gruber S., Huiban M., Passchier J., Mercier J., Génicot C., Gouverneur V. (2014). A General Copper-Mediated Nucleophilic ^18^F Fluorination of Arenes. Angew. Chem. Int. Ed..

[28] Zlatopolskiy B.D., Zischler J., Krapf P., Zarrad F., Urusova E.A., Kordys E., Endepols H., Neumaier B. (2015). Copper-Mediated Aromatic Radiofluorination Revisited: Efficient Production of PET Tracers on a Preparative Scale. Chem. Eur. J..

[29] Mossine A.V., Brooks A.F., Makaravage K.J., Miller J.M., Ichiishi N., Sanford M.S., Scott P.J.H. (2015). Synthesis of [^18^F]Arenes via the Copper-Mediated [^18^F]Fluorination of Boronic Acids. Org. Lett..

[30] Vitaku E., Smith D.T., Njardarson J.T. (2014). Analysis of the Structural Diversity, Substitution Patterns, and Frequency of Nitrogen Heterocycles among U.S. FDA Approved Pharmaceuticals. J. Med. Chem..

[31] Altıntop M.D., Sever B., Akalın Çiftçi G., Kucukoglu K., Özdemir A., Soleimani S.S., Nadaroglu H., Kaplancıklı Z.A. (2017). Synthesis and evaluation of new benzodioxole-based dithiocarbamate derivatives as potential anticancer agents and hCA-I and hCA-II inhibitors. Eur. J. Med. Chem..

[32] Naim M.J.N., Alam O., Alam M.J., Alam P., Shrivastava N. (2015). A review on pharmacological profile of Morpholine derivatives. Int. J. Pharm. Pharm. Sci..

[33] Raju G.N., Suresh P.V., Nadendla R.R., Anusha K. (2015). Synthesis, characterization and antimicrobial evaluation of isoxazole derivatives. Der. Pharm. Chem..

[34] Swellmeen L. (2016). 1,3-Oxazole Derivatives: A Review of Biological Activities as Antipathogenic. Der. Pharm. Chem..

[35] Joshi S., Bisht A.S., Juyal D. (2017). Systematic scientific study of 1, 3-oxazole derivatives as a useful lead for pharmaceuticals: A review. J. Pharm. Innov..

[36] Reza Kazemizadeh A., Ramazani A. (2012). Synthetic Applications of Passerini Reaction. Curr. Org. Chem..

