# Supplementary material for: MCR Scaffolds Get Hotter with 18F-Labeling
Source: Molecules. 2019 Apr 4;24(7):1327. doi: 10.3390/molecules24071327 (PMC6480256; doi:10.3390/molecules24071327)

SUPPORTING INFORMATION

MCR scaffolds get hotter with ^{18}- Labeling

Tryfon Zarganes-Tzitzikas [1,†], Gonçalo S. Clemente [2,†], Philip H. Elsinga [2,*] and Alexander Dömling [1,*]

[1] Department of Drug Design, Groningen Research Institute of Pharmacy, University of Groningen, The Netherlands

[2] Department of Nuclear Medicine and Molecular Imaging, University Medical Center Groningen, University of Groningen, The Netherlands

* Correspondence: p.h.elsinga@umcg.nl (P.H.E); a.s.s.domling@rug.nl.com (A.D.)

† These authors contributed equally to this paper.

Table of contents

Figure S1 to Figure S13: Examples of the radio-TLCs 2

Table S1. TLC-SG retention factor (Rf) profiles for the tested compounds 8

Figure S14: Effect of temperature in RCC of **[^{18}F]1** 9

Scheme S1: Recent late-stage strategies for the ^{18}F-fluorination of (hetero)arenes 10

Scheme S2: Proposed mechanism for copper-mediated oxidative ^{18}F-fluorination of aryl boronic esters 11

Spectra of compounds 12

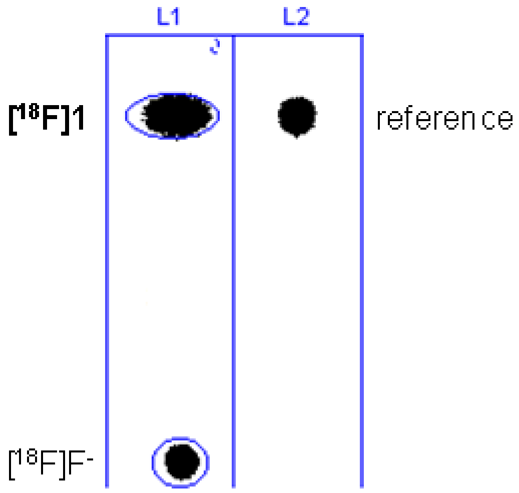

Figure S1. Example of the radio-TLC profile of 4-[^{18}F]fluorobenzaldehyde **[^{18}F]1**.

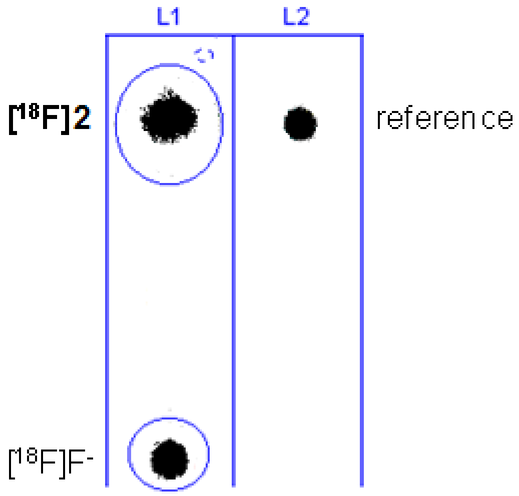

Figure S2. Example of the radio-TLC profile of 4-[^{18}F]fluorobenzonitrile **[^{18}F]2**.

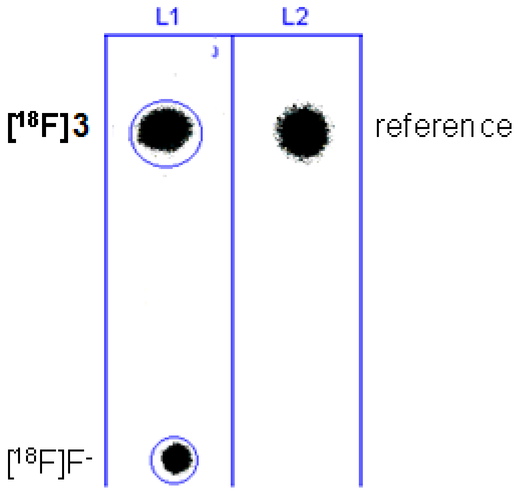

Figure S3. Example of the radio-TLC profile of 4-[^{18}F]fluoro-1,2-dimethoxybenzene **[^{18}F]3**.

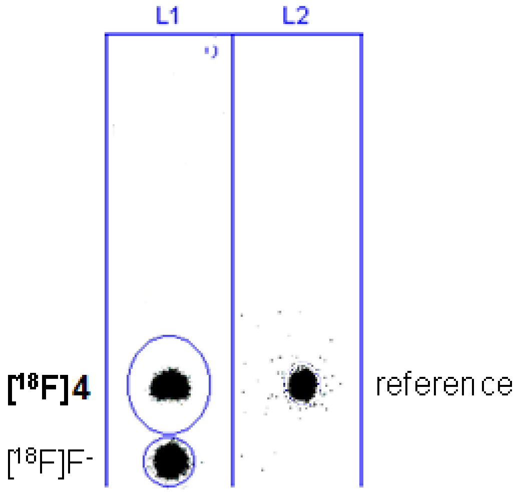

Figure S4. Example of the radio-TLC profile of *N*-(tert-butyl)-2-(4-[^{18}F]fluorophenyl)-2-(2-oxoazetidin-1-yl)acetamide **[^{18}F]4**.

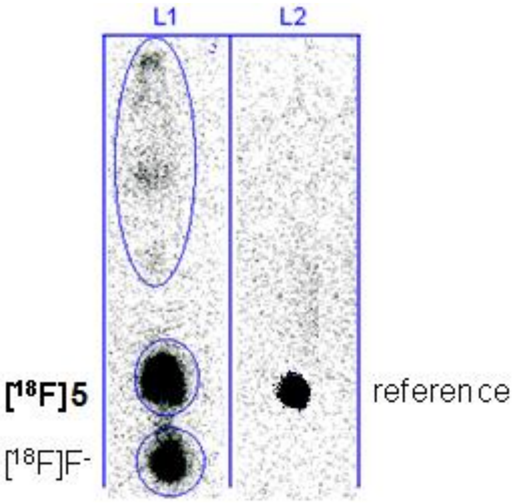

Figure S5. Example of the radio-TLC profile of *N*-(4-bromobenzyl)-2-(4-[18F]fluorophenyl)-2-(2-oxoazetidin-1-yl) **[18F]5**.

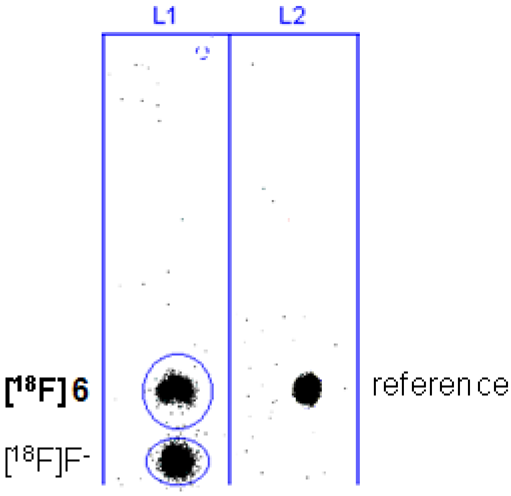

Figure S6. Example of the radio-TLC profile of 4-((1-(benzo[d][1,3]dioxol-5-ylmethyl)-1H-tetrazol-5-yl)(4-[18F]fluorophenyl)methyl)morpholine **[18F]6**.

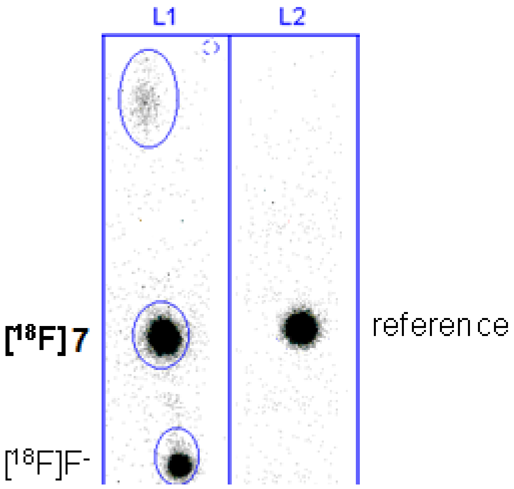

Figure S7. Example of the radio-TLC profile of 4-((1-(2,6-dimethylphenyl)-1H-tetrazol-5-yl)(4-[^{18}F]fluorophenyl)methyl)morpholine **[^{18}F]7**.

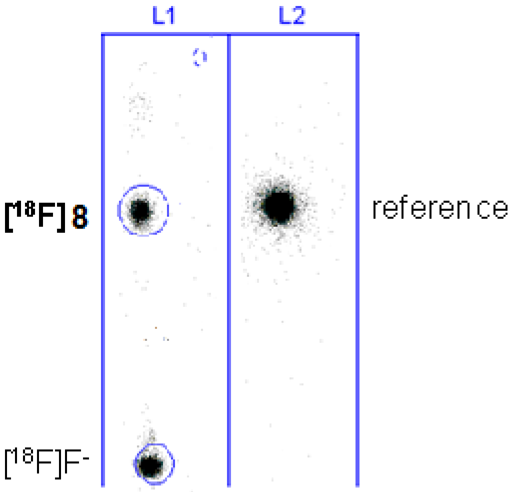

Figure S8. Example of the radio-TLC profile of 1-(1-benzyl-1H-tetrazol-5-yl)-N-(4-chlorobenzyl)-1-(4-[^{18}F]fluorophenyl)methanamine **[^{18}F]8**.

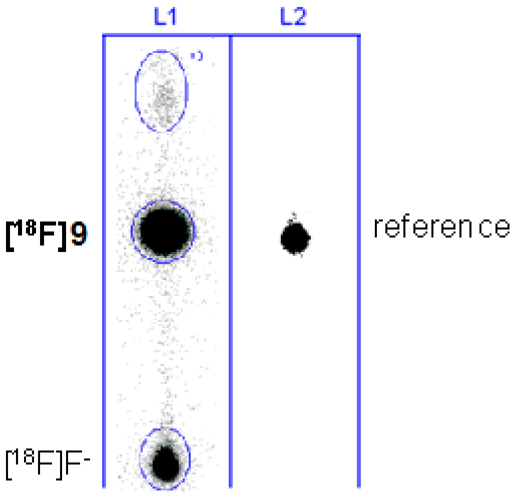

Figure S9. Example of the radio-TLC profile of 5-(4-[^{18}F]fluorophenyl)oxazole **[^{18}F]9**.

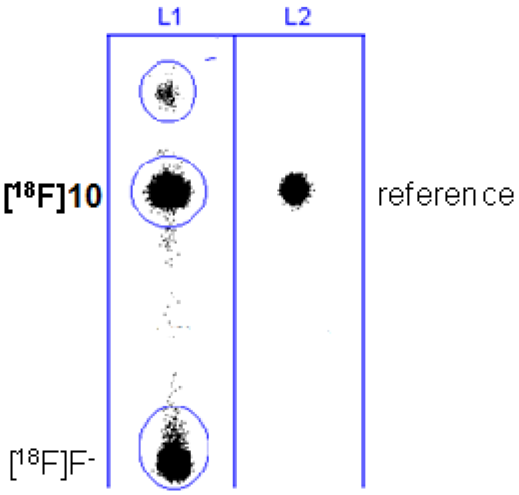

Figure S10. Example of the radio-TLC profile of 5-(3-[^{18}F]fluorophenyl)oxazole **[^{18}F]10**.

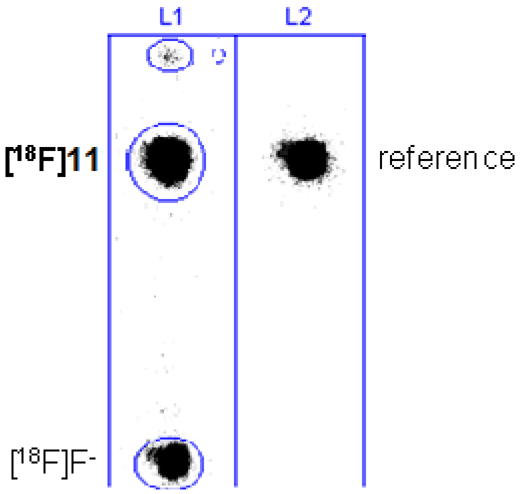

Figure S11. Example of the radio-TLC profile of 1-(4-[^{18}F]fluorophenyl)-2-oxo-2-((3,4,5-trimethoxybenzyl)amino)ethyl 3-bromo-2-methylbenzoate **[^{18}F]11**.

.

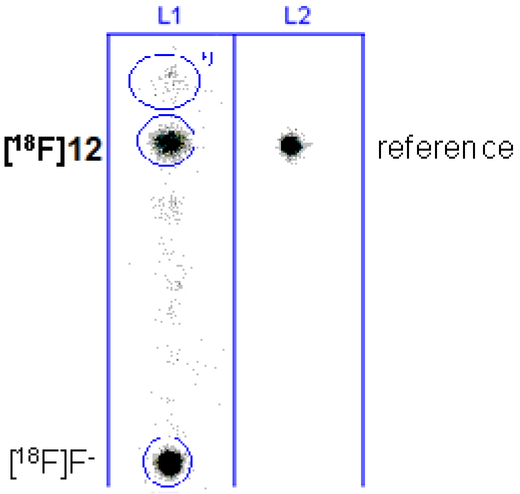

Figure S12. Example of the radio-TLC profile of 2-((benzo[d][1,3]dioxol-5-ylmethyl)amino)-1-(4-[^{18}F]fluorophenyl)-2-oxoethyl 2,4-dimethylbenzoate **[^{18}F]12**

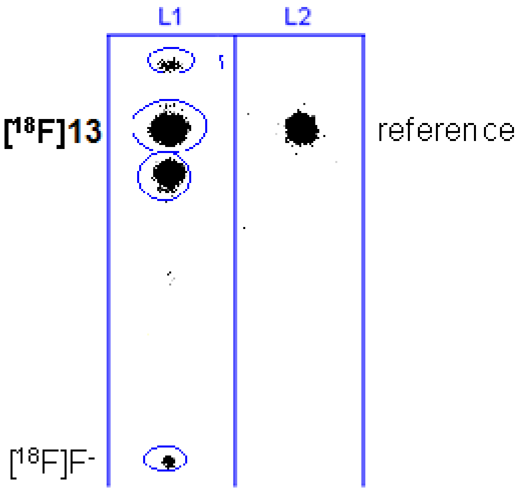

Figure S13. Example of the radio-TLC profile of 2-(benzylamino)-1-(4-[^{18}F]fluorophenyl)-2-oxoethyl cyclohexanecarboxylate **[^{18}F]13**.

Table S1. TLC-SG retention factor (Rf) profiles for the tested compounds

Compound	R_f	Hexane:ethyl acetate ratio
**[18/19F]1**	0.85	2:1
**[18/19F]2**	0.85	2:1
**[18/19F]3**	0.80	2:1
**[18/19F]4**	0.40	1:1
**[18/19F]5**	0.30	1:1
**[18/19F]6**	0.35	2:1
**[18/19F]7**	0.40	2:1
**[18/19F]8**	0.60	2:1
**[18/19F]9**	0.50	2:1
**[18/19F]10**	0.65	2:1
**[18/19F]11**	0.75	2:1
**[18/19F]12**	0.8	2:1
**[19F]13**	0.85	2:1
[18F]F^-	0.0	1:1; 2:1
copper–[18F]fluoride complex	1.0	1:1; 2:1

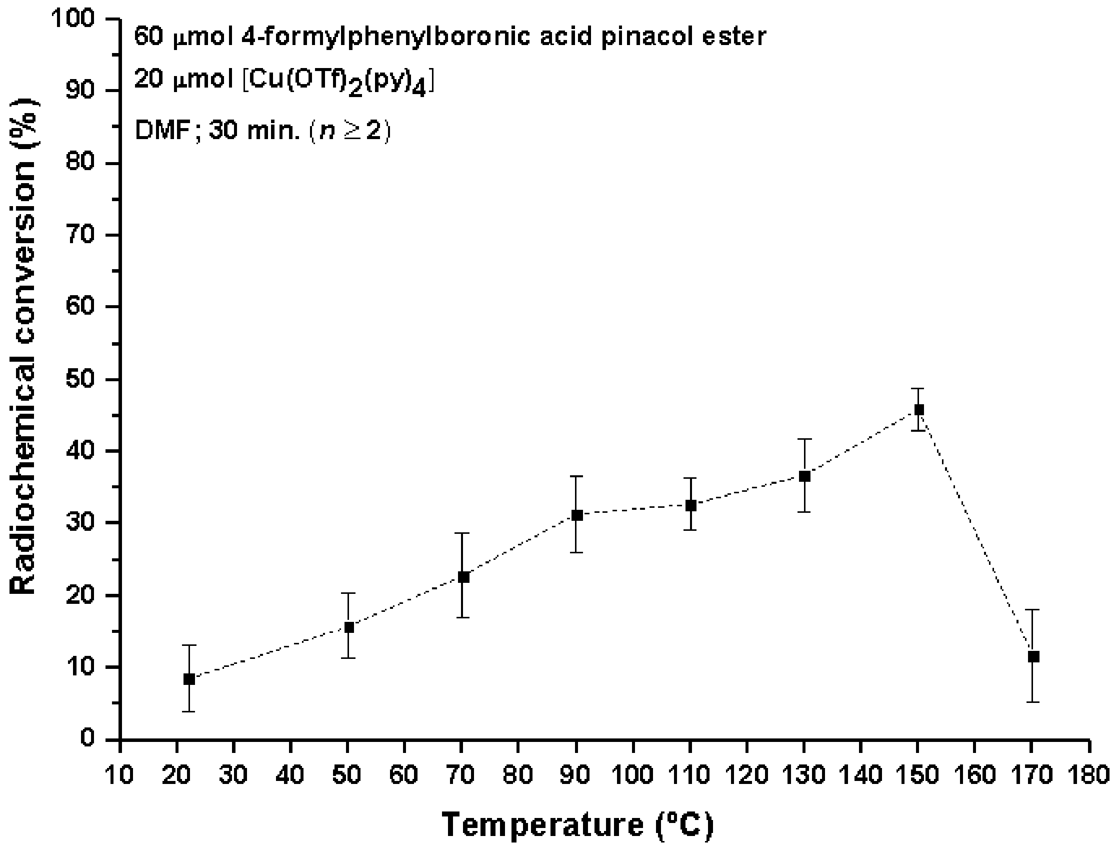

Figure S14. Effect of temperature in RCC of **[18F]1**.

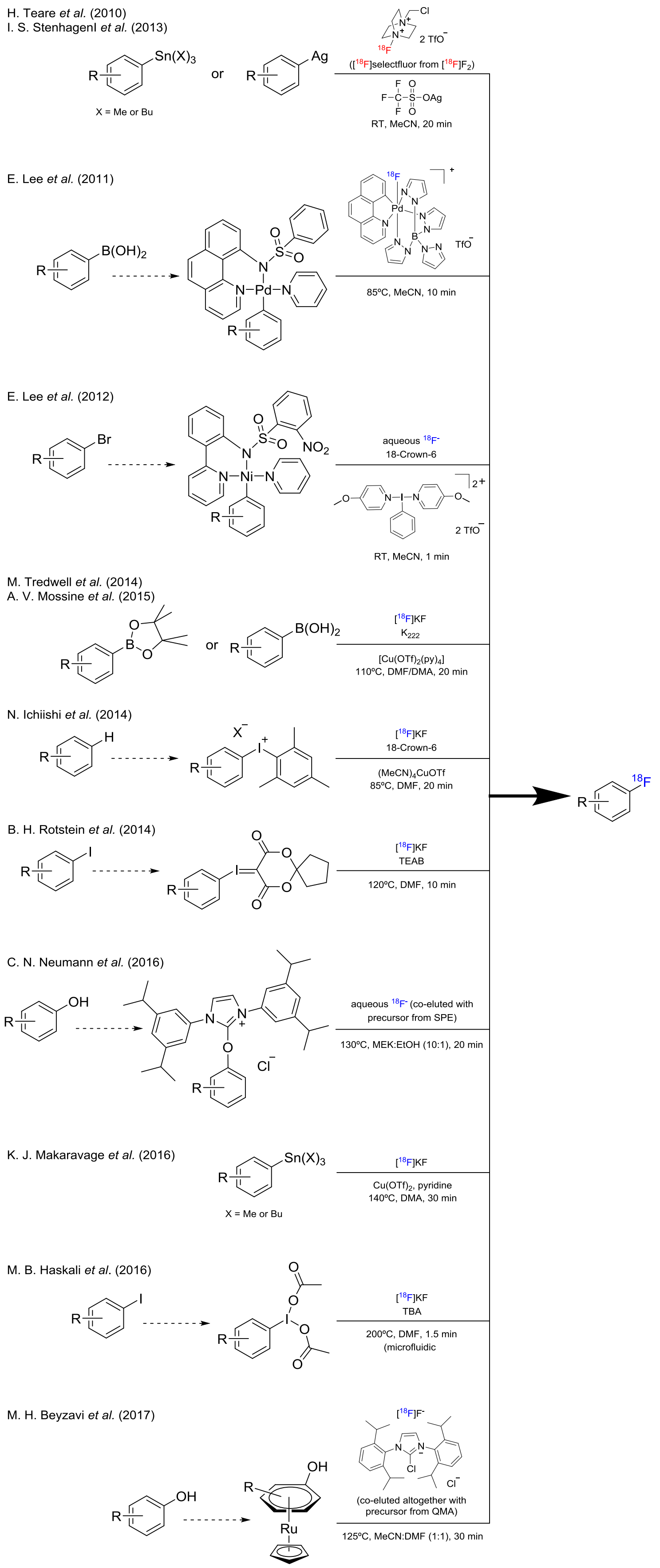

Scheme S1. Recent late-stage strategies for the ^{18}F-fluorination of (hetero)arenes.

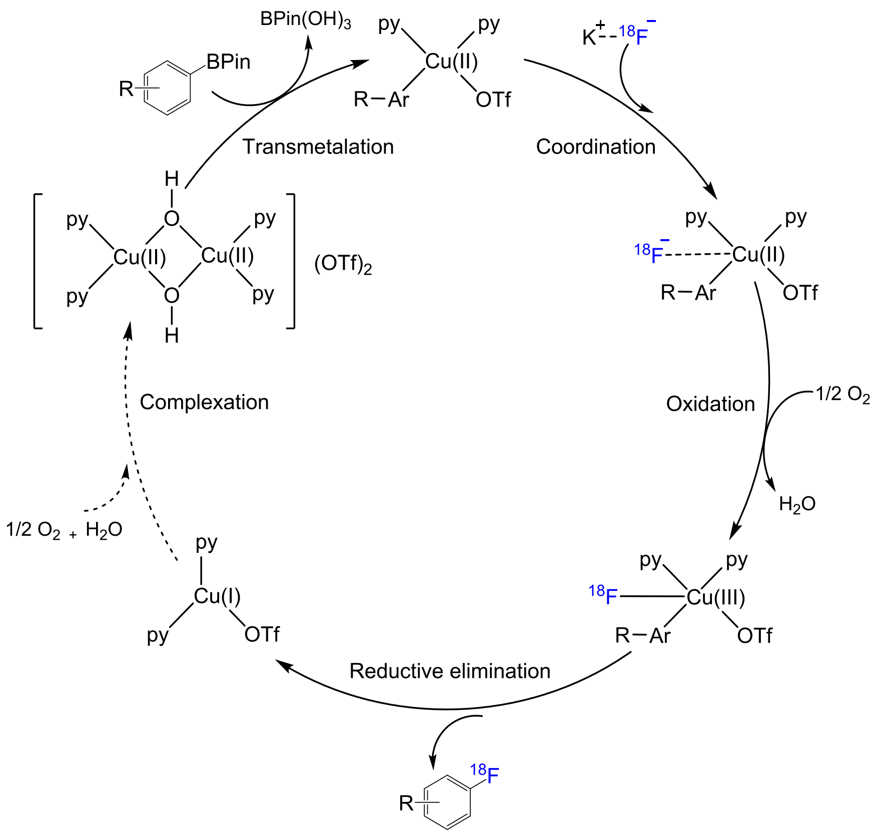

Scheme S2. Proposed mechanism for copper-mediated oxidative ^{18}F-fluorination of aryl boronic esters.

Spectra of compounds

***N*-(tert-butyl)-2-(2-oxoazetidin-1-yl)-2-(4-(4,4,5,5-tetramethyl-1,3,2-dioxaborolan-2-yl)phenyl)acetamide (4)**:

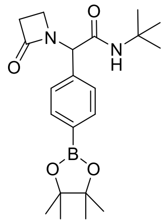

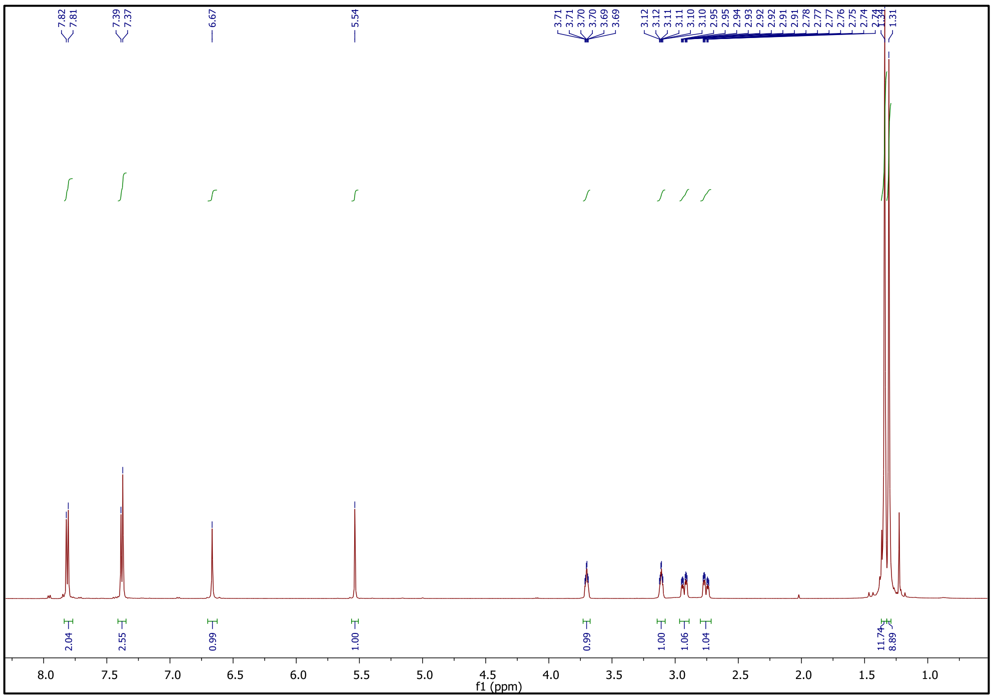

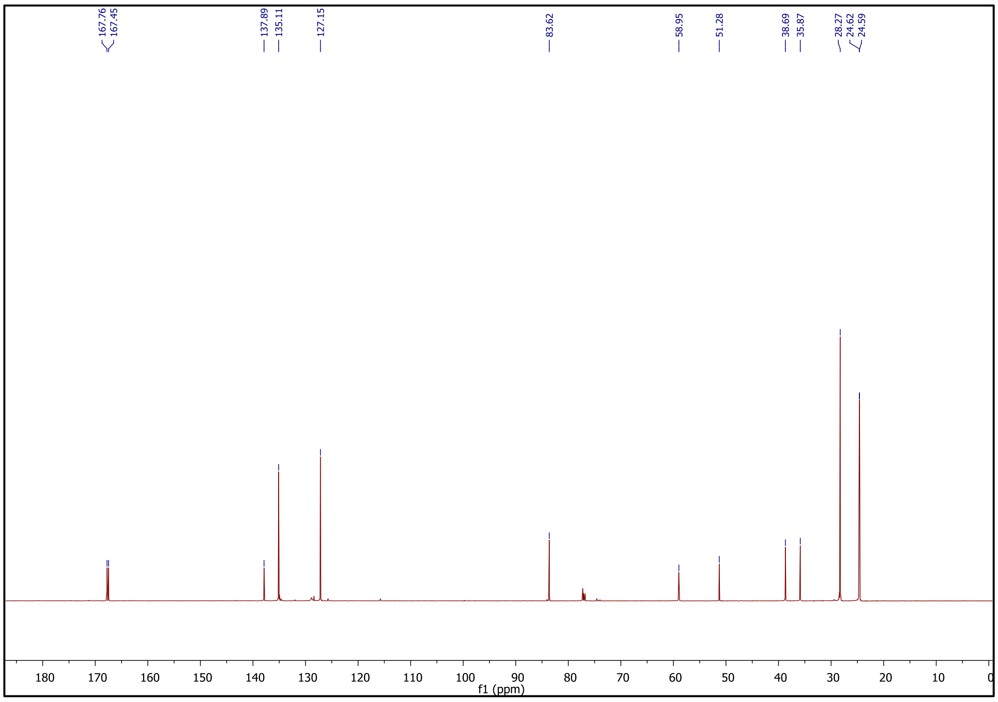

N-(tert-butyl)-2-(4-fluorophenyl)-2-(2-oxoazetidin-1-yl)acetamide ([19F]4):

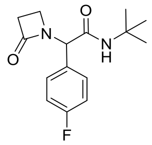

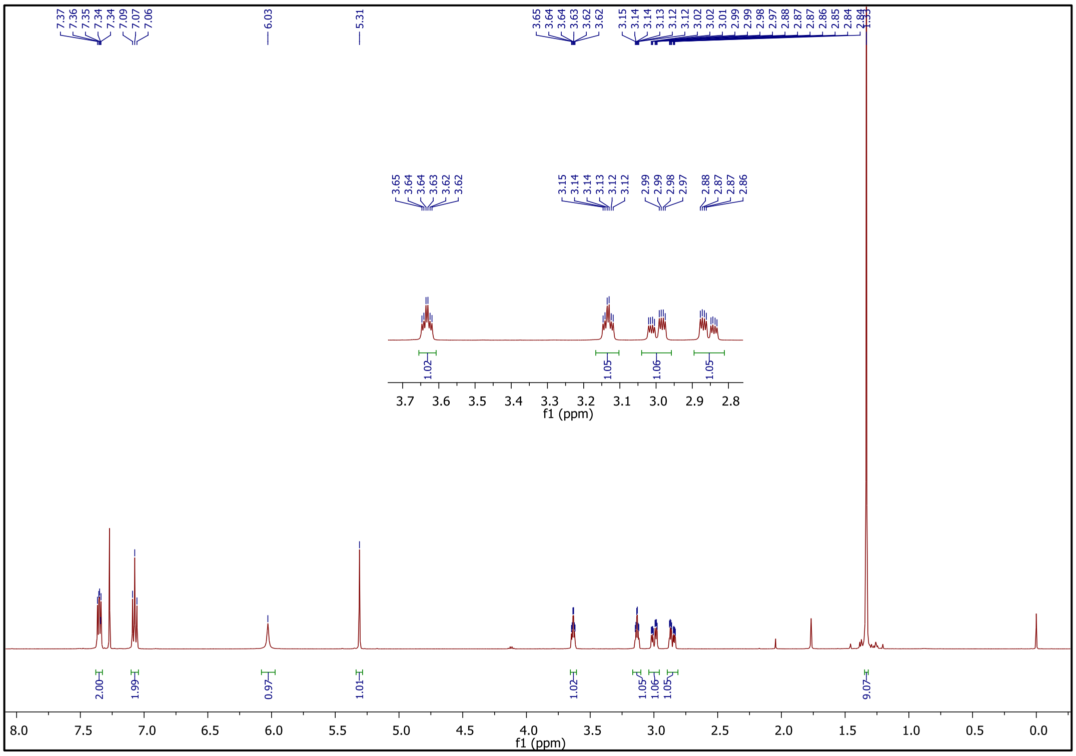

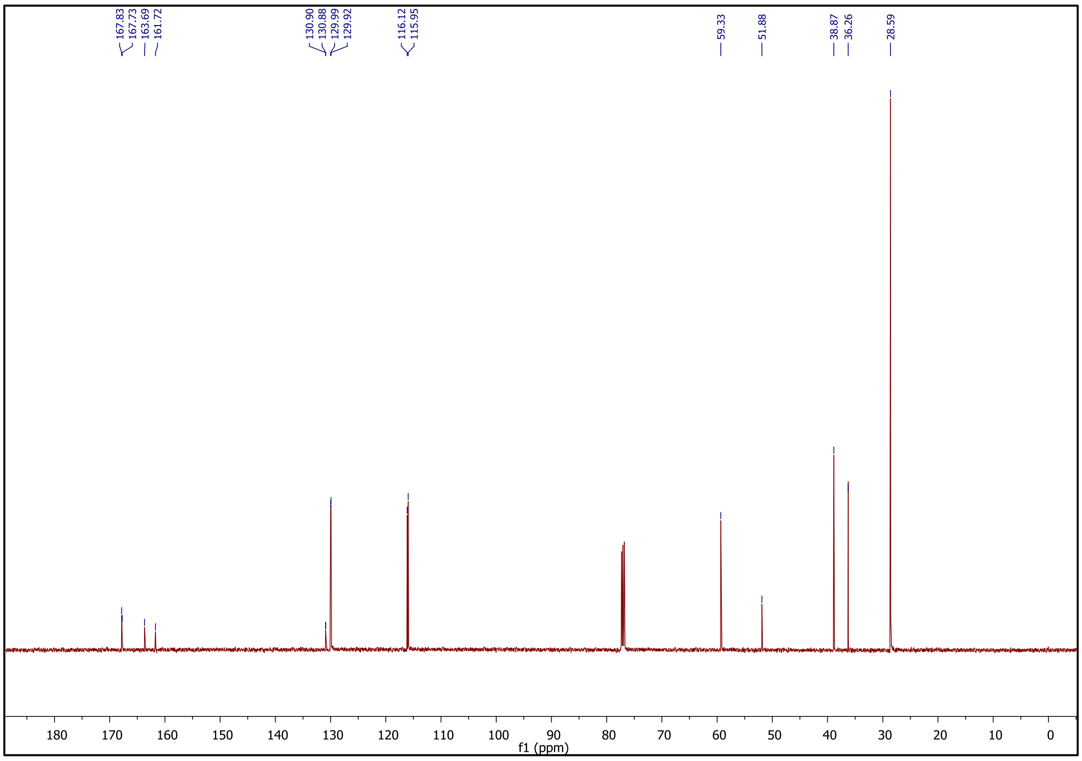

N-(4-bromobenzyl)-2-(2-oxoazetidin-1-yl)-2-(4-(4,4,5,5-tetramethyl-1,3,2-dioxaborolan-2-yl)phenyl)acetamide (5):

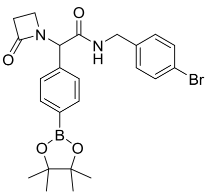

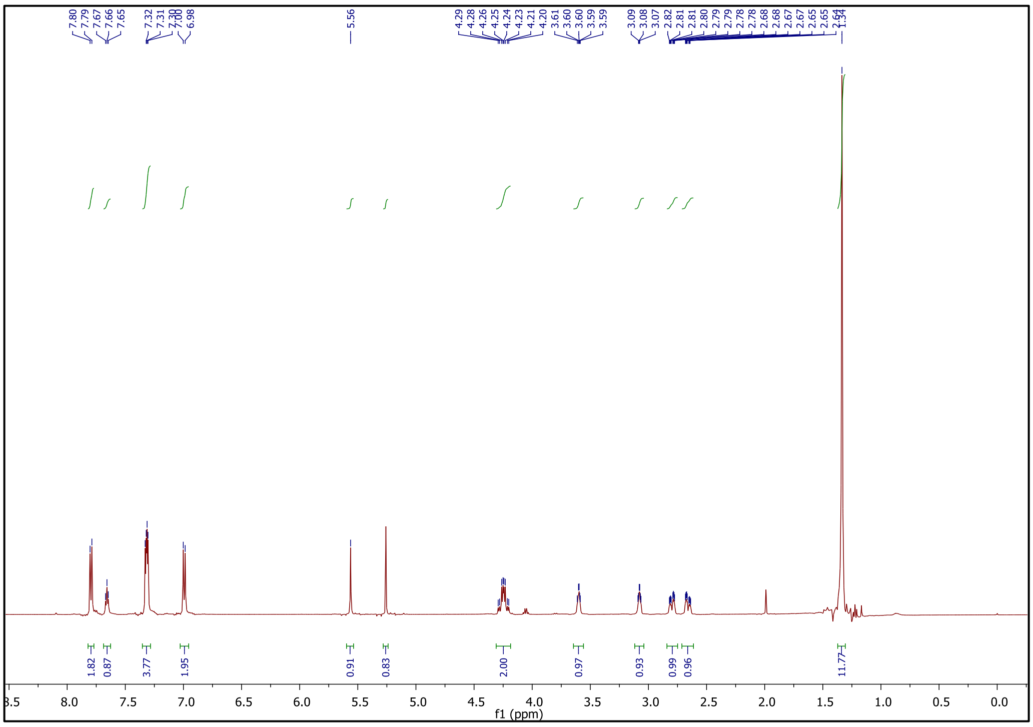

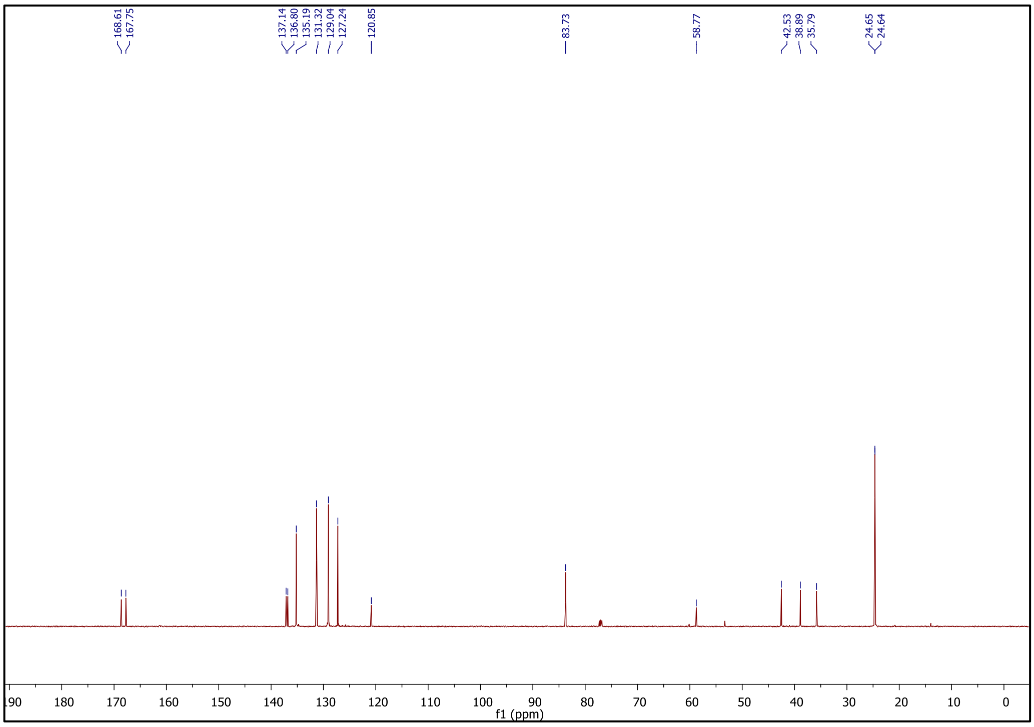

***N*-(4-bromobenzyl)-2-(4-fluorophenyl)-2-(2-oxoazetidin-1-yl) ([19F]5):**

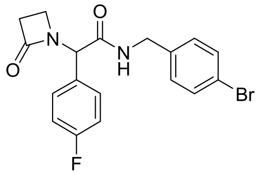

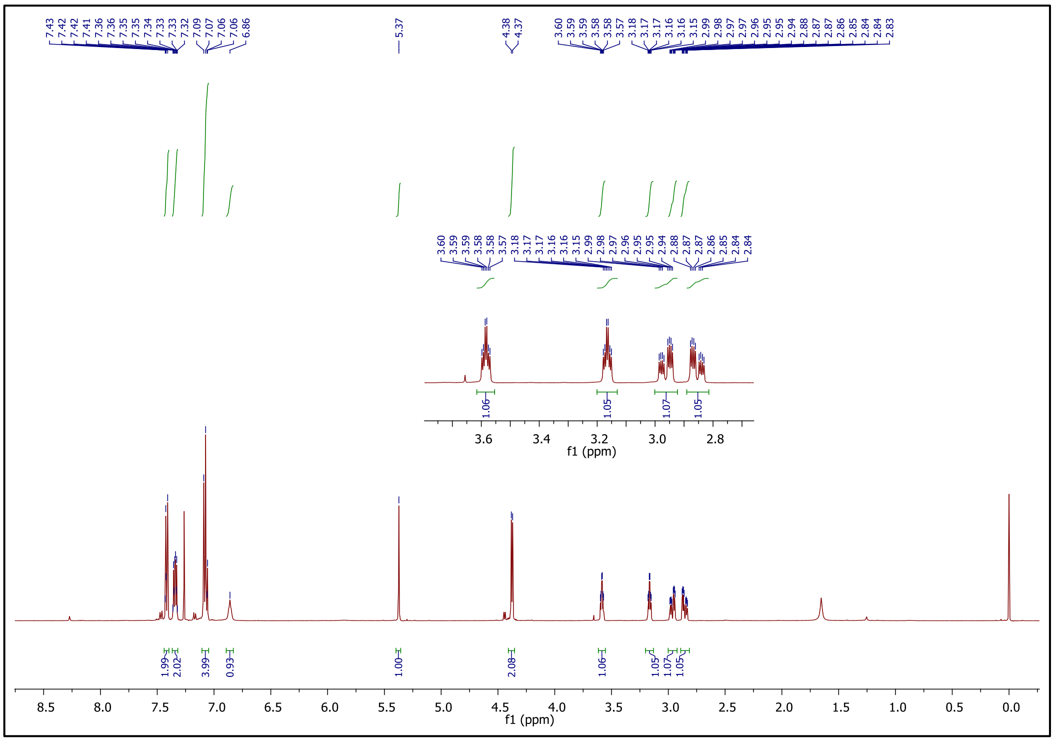

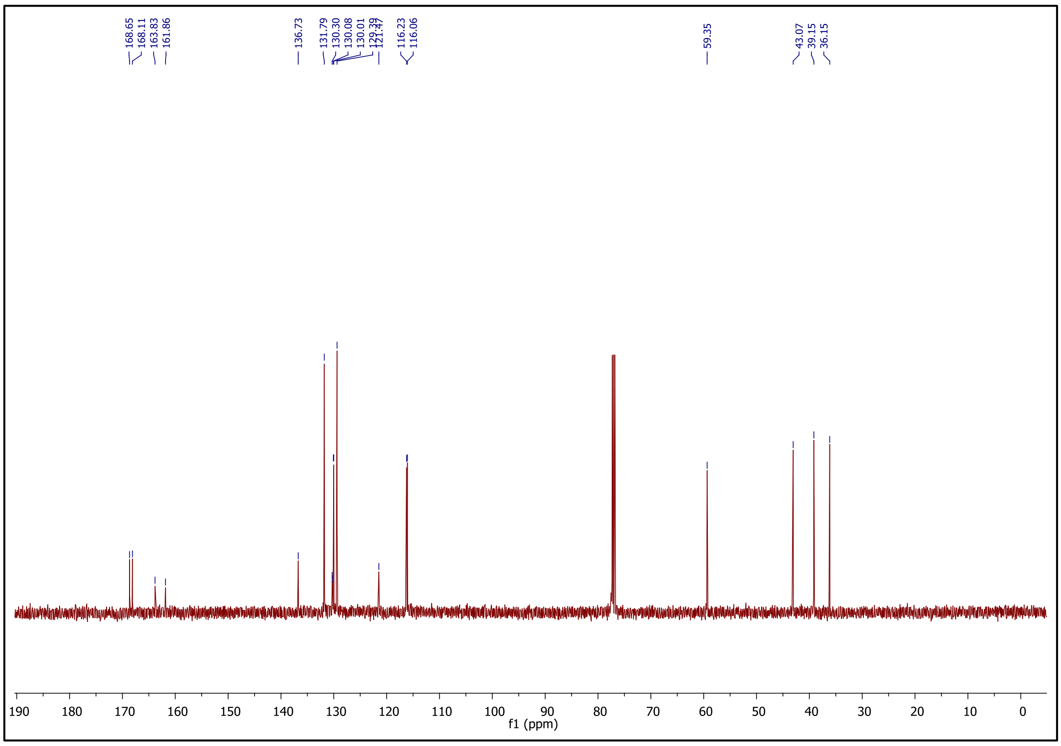

4-((1-(benzo[d][1,3]dioxol-5-ylmethyl)-1H-tetrazol-5-yl)(4-(4,4,5,5-tetramethyl-1,3,2-dioxaborolan-2-yl)phenyl)methyl)morpholine (6):

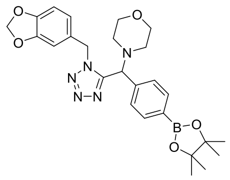

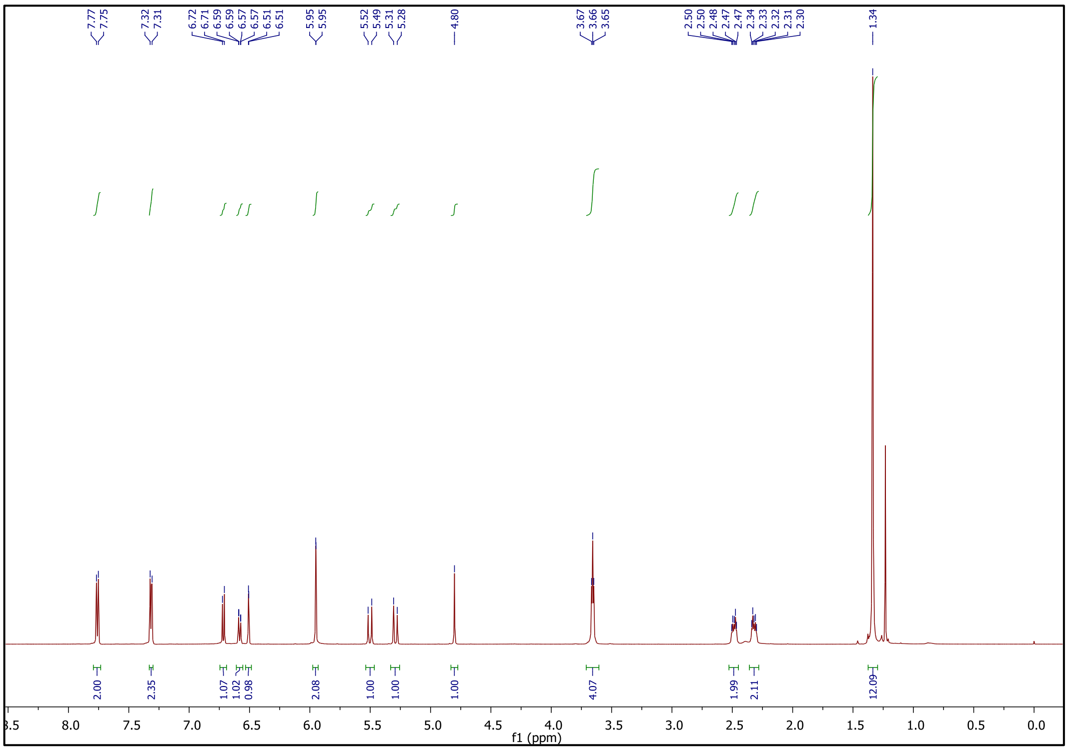

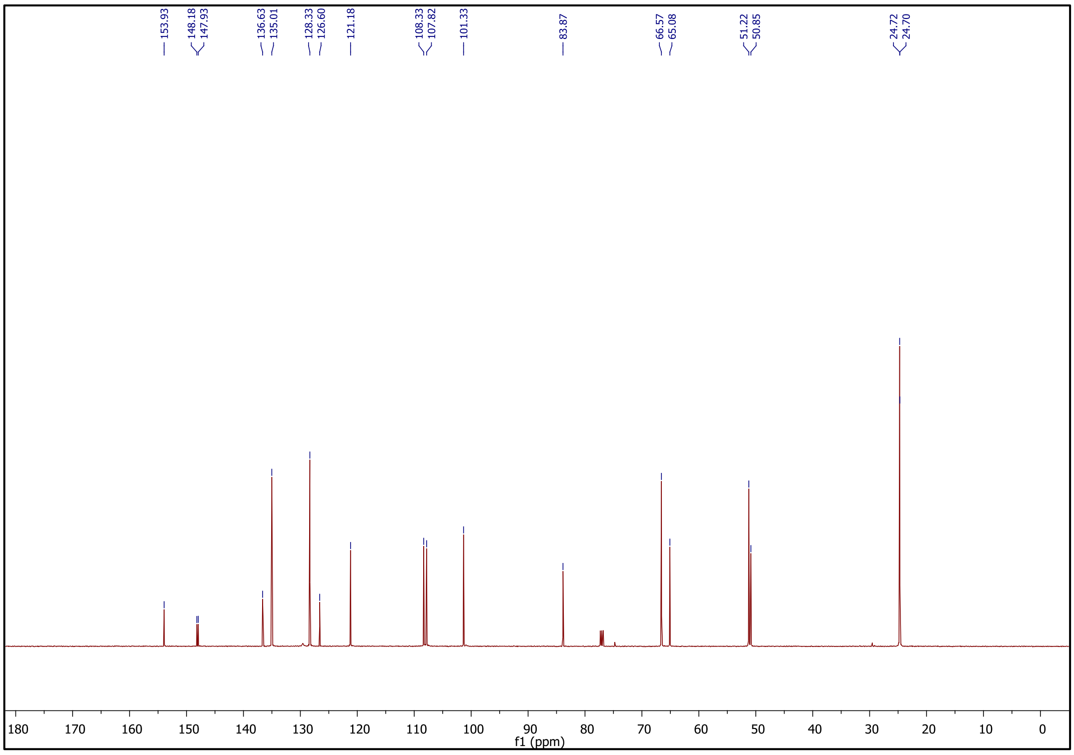

4-((1-(benzo[d][1,3]dioxol-5-ylmethyl)-1H-tetrazol-5-yl)(4-fluorophenyl)methyl)morpholine ([19F]6):

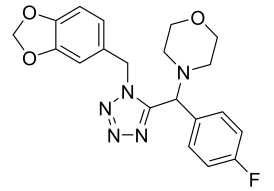

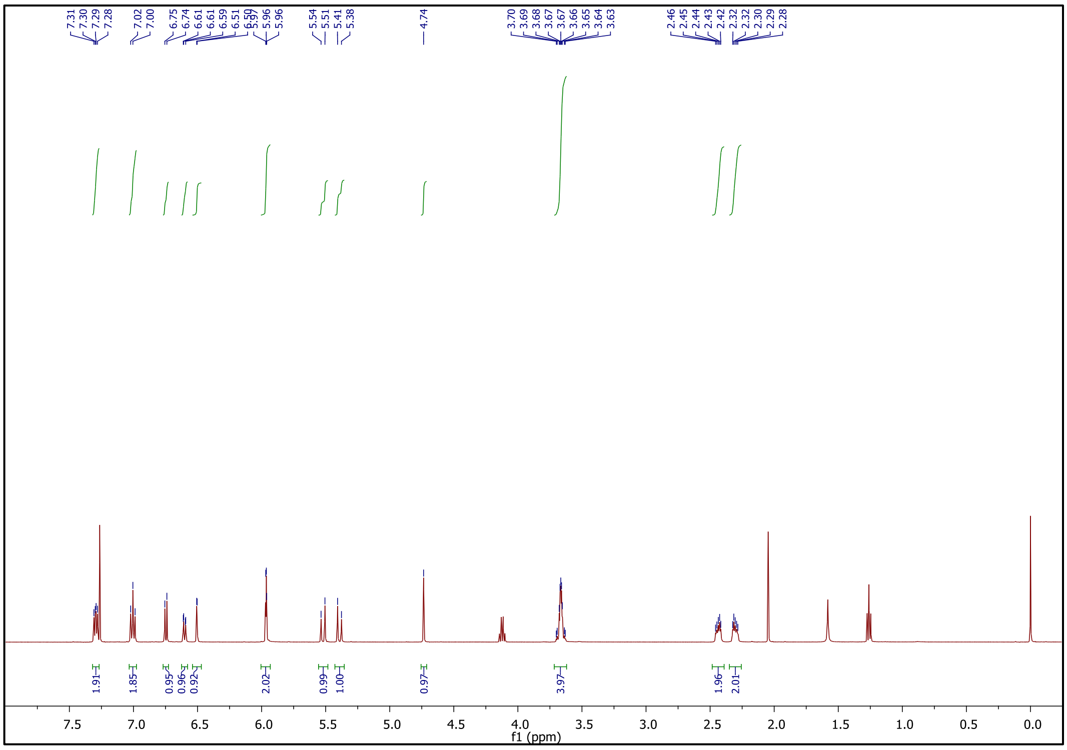

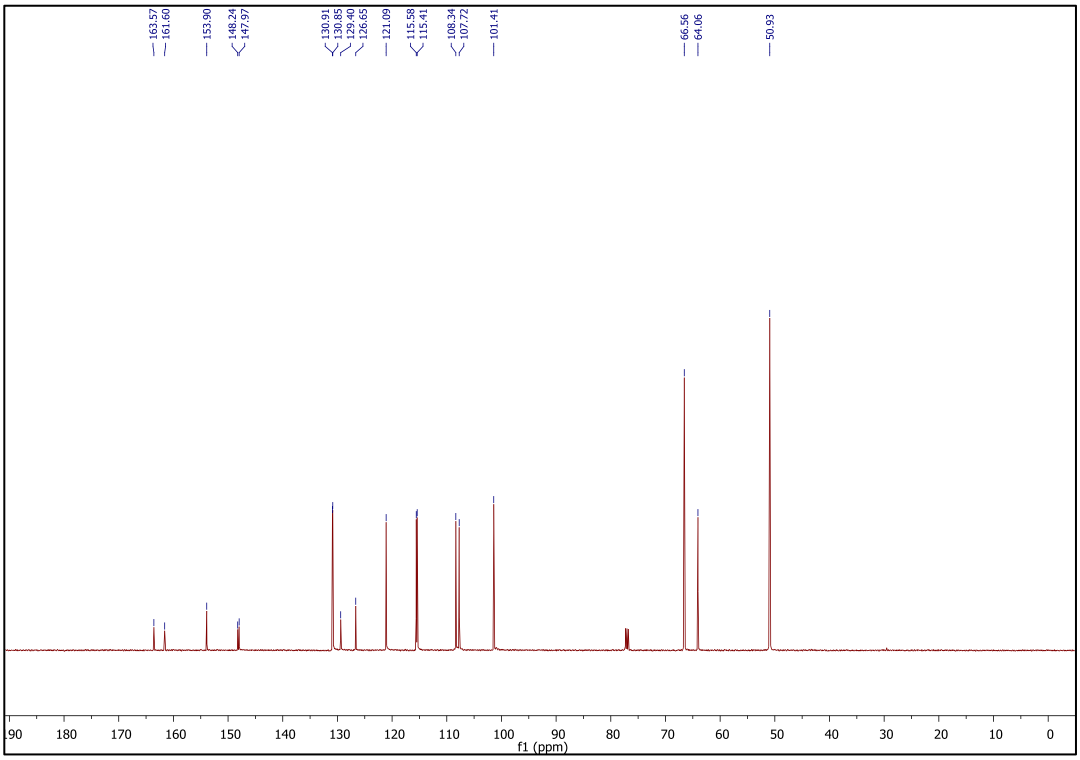

4-((1-(2,6-dimethylphenyl)-1H-tetrazol-5-yl)(4-(4,4,5,5-tetramethyl-1,3,2-dioxaborolan-2-yl)phenyl)methyl)morpholine (7):

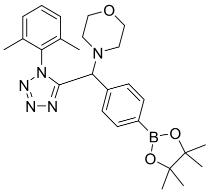

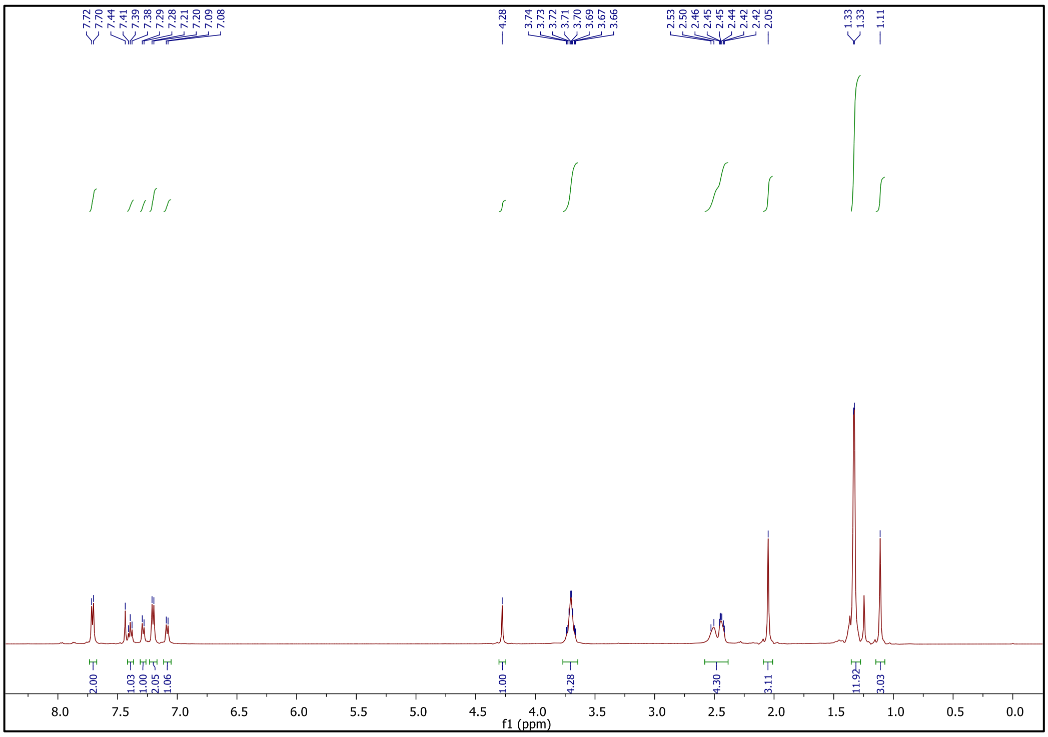

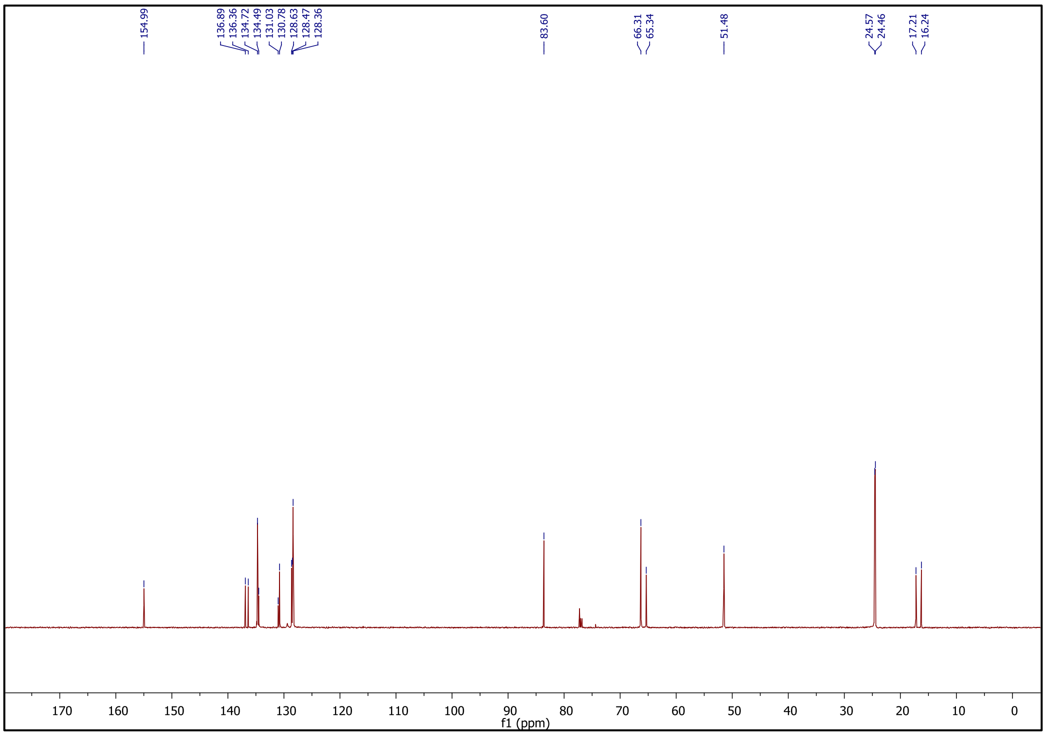

4-((1-(2,6-dimethylphenyl)-1H-tetrazol-5-yl)(4-fluorophenyl)methyl)morpholine ([^{19}F]7):

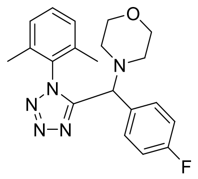

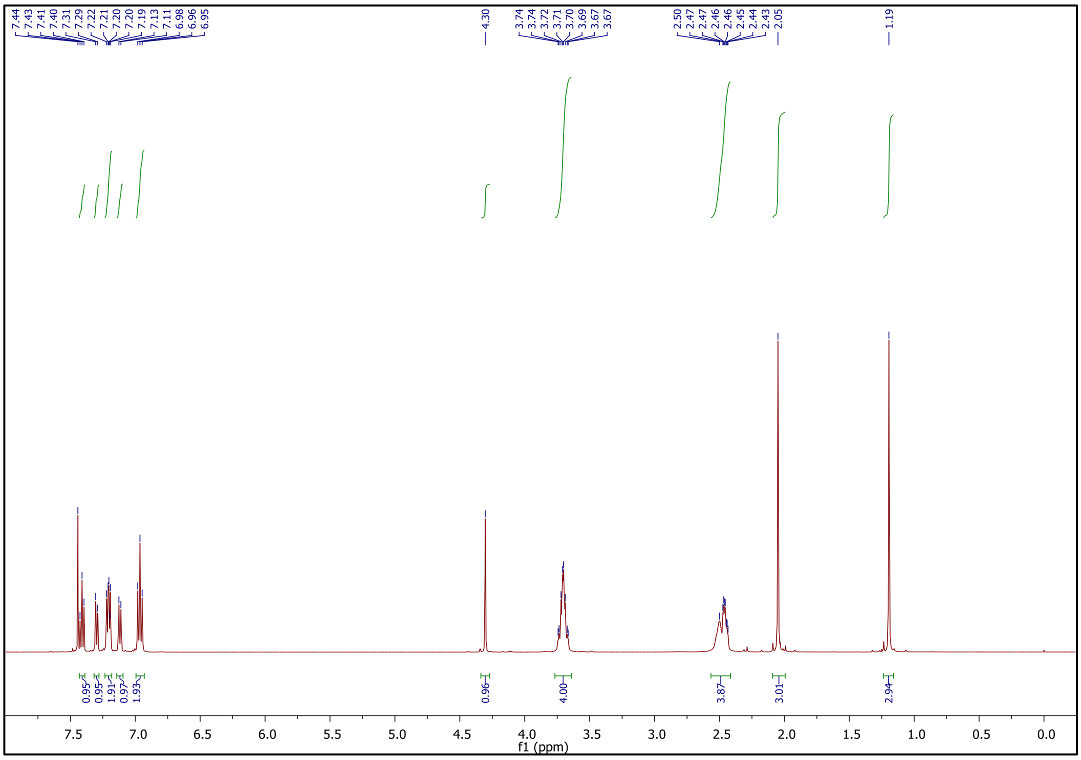

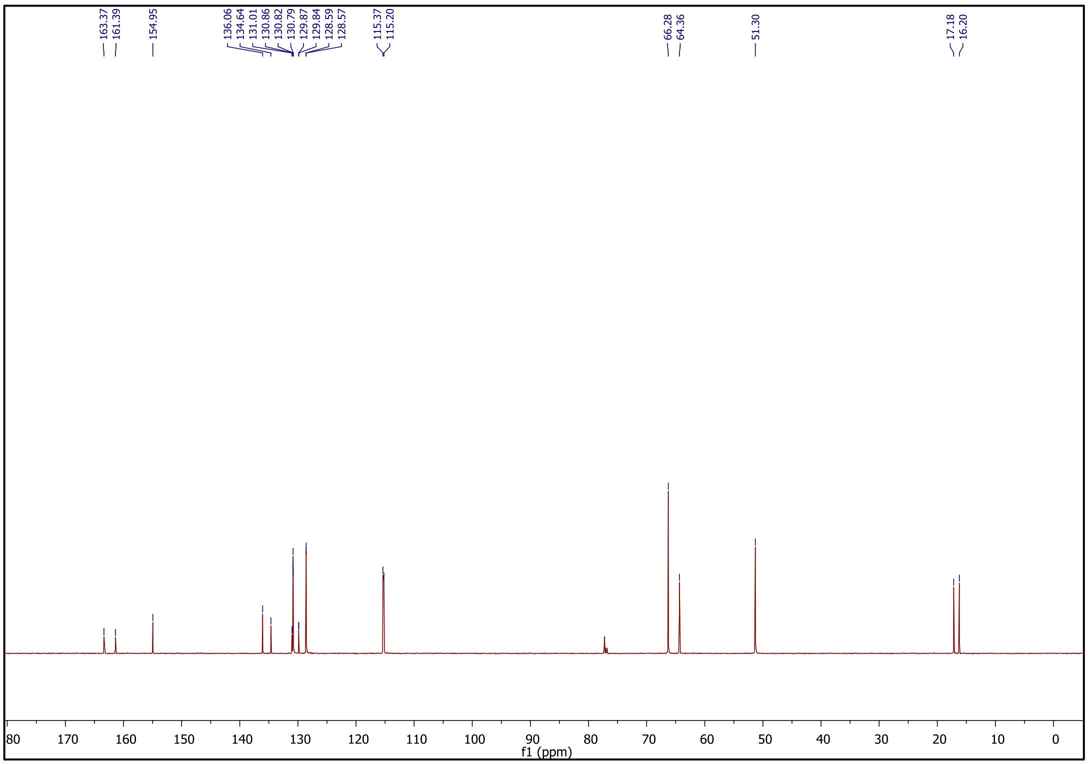

1-(1-benzyl-1H-tetrazol-5-yl)-N-(4-chlorobenzyl)-1-(4-(4,4,5,5-tetramethyl-1,3,2-dioxaborolan-2-yl)phenyl)methanamine (8):

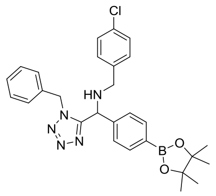

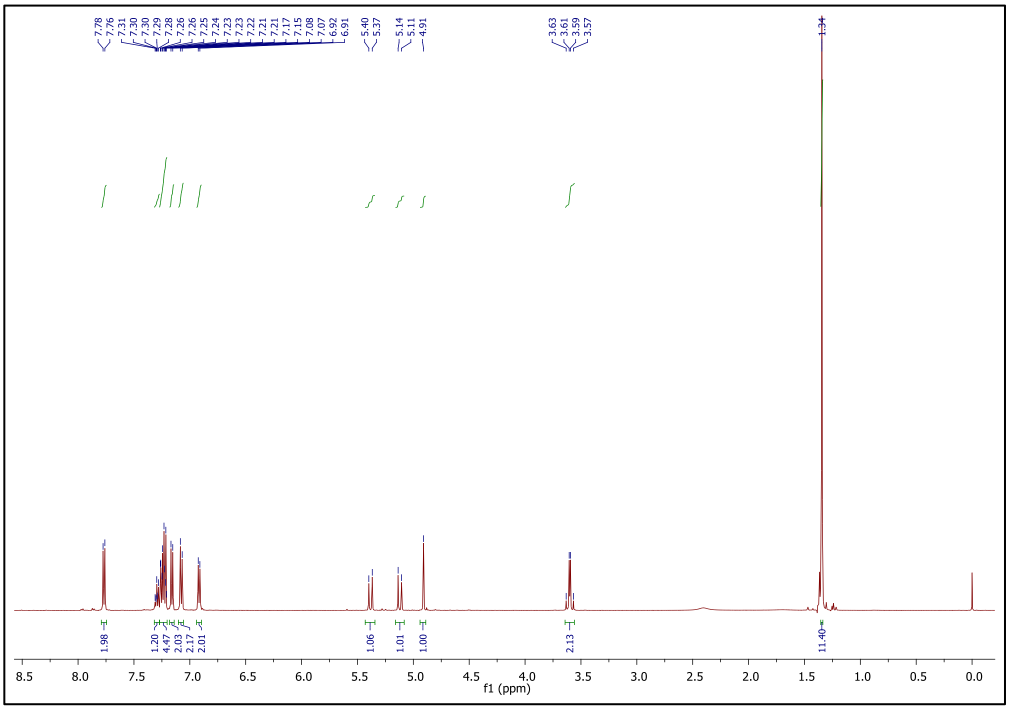

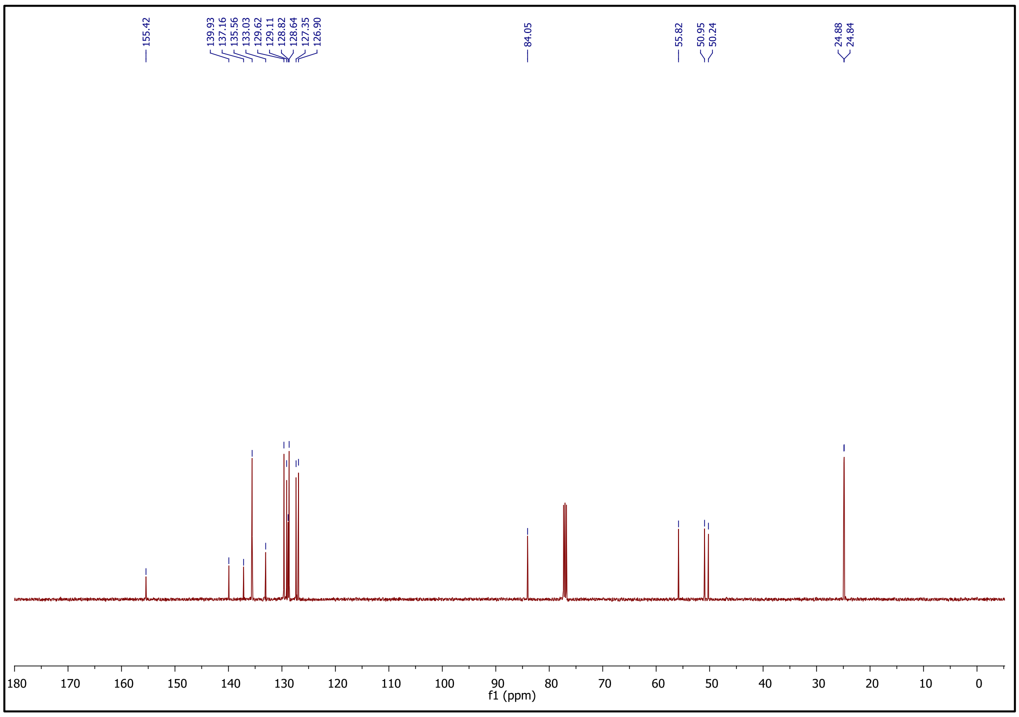

1-(1-benzyl-1H-tetrazol-5-yl)-N-(4-chlorobenzyl)-1-(4-fluorophenyl)methanamine ([^{19}F]8):

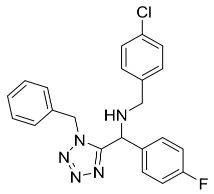

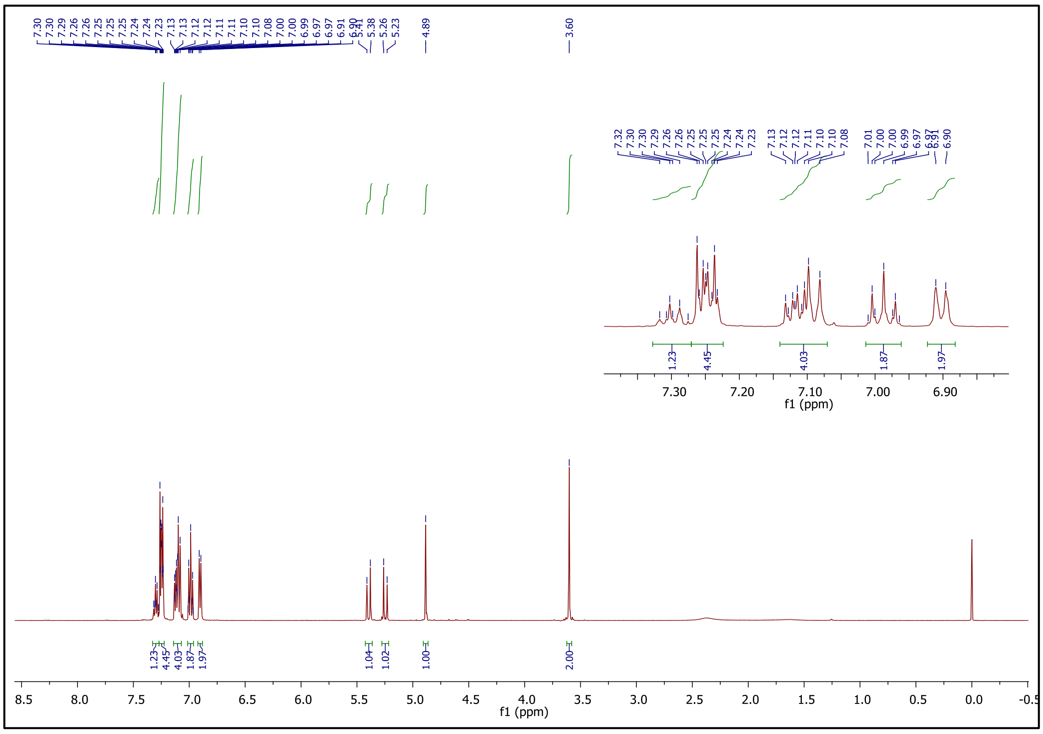

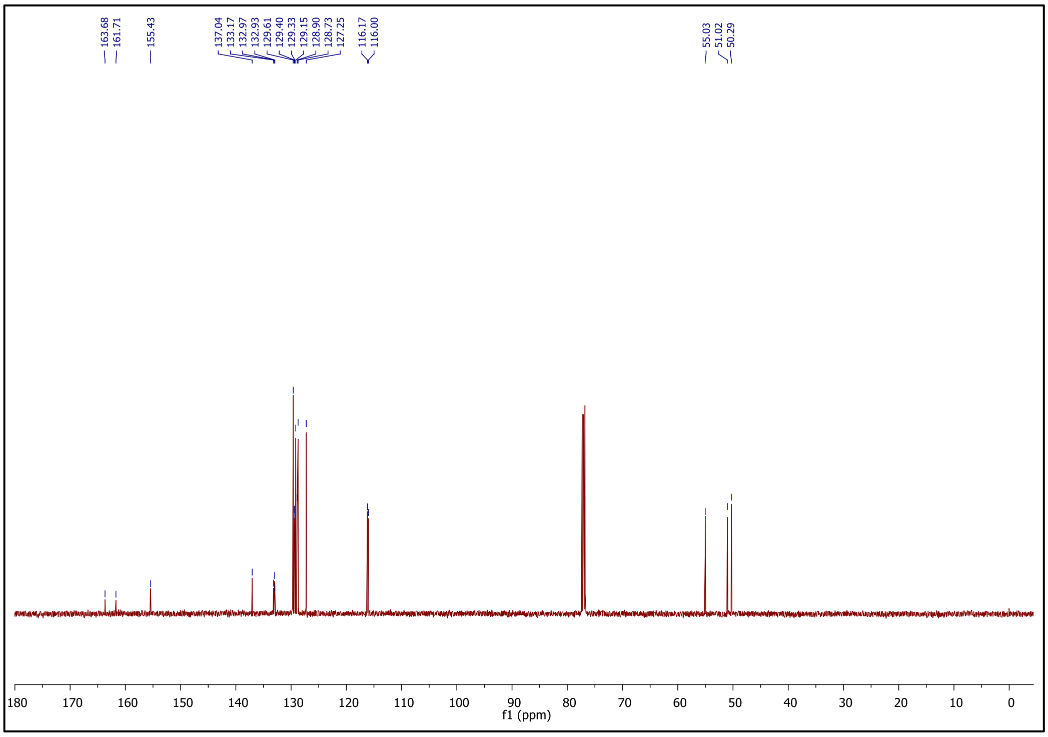

5-(4-(4,4,5,5-tetramethyl-1,3,2-dioxaborolan-2-yl)phenyl)oxazole (9):

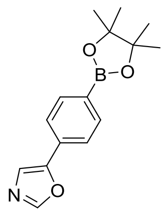

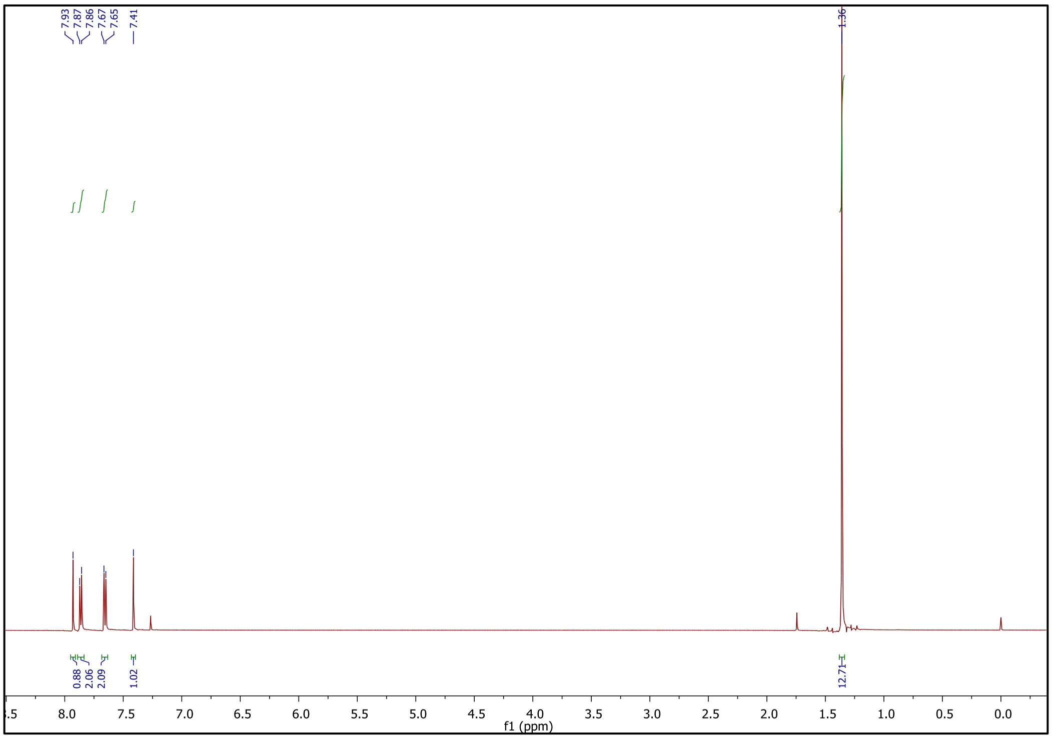

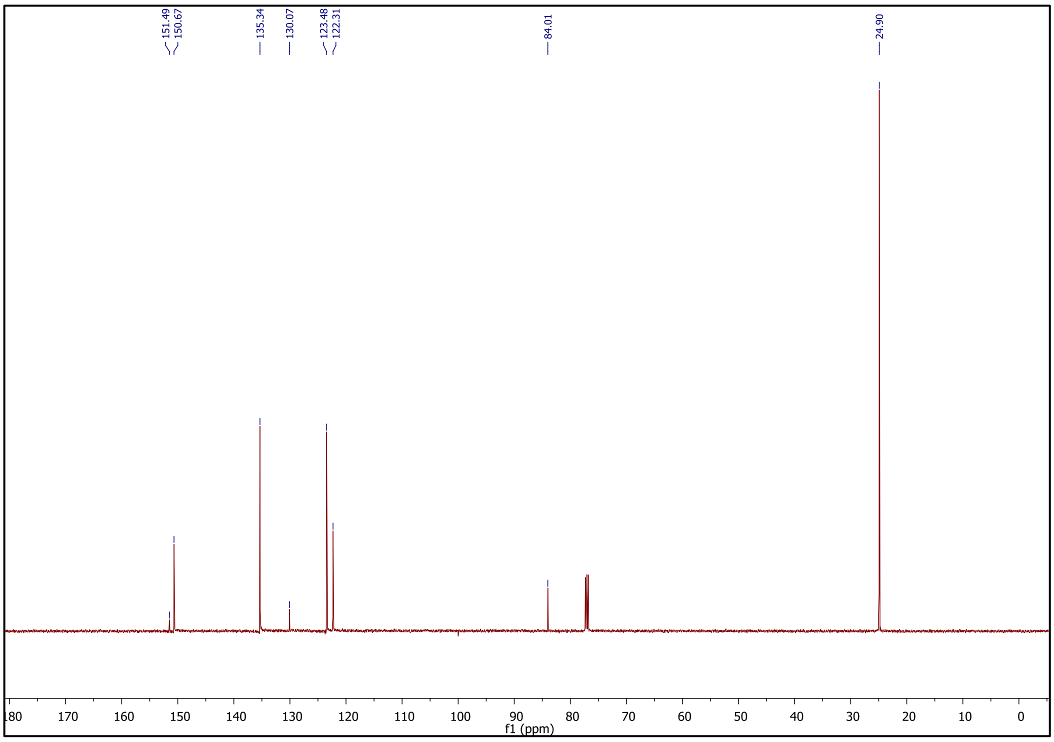

5-(4-fluorophenyl)oxazole ([19F]9):

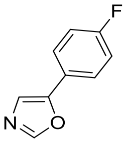

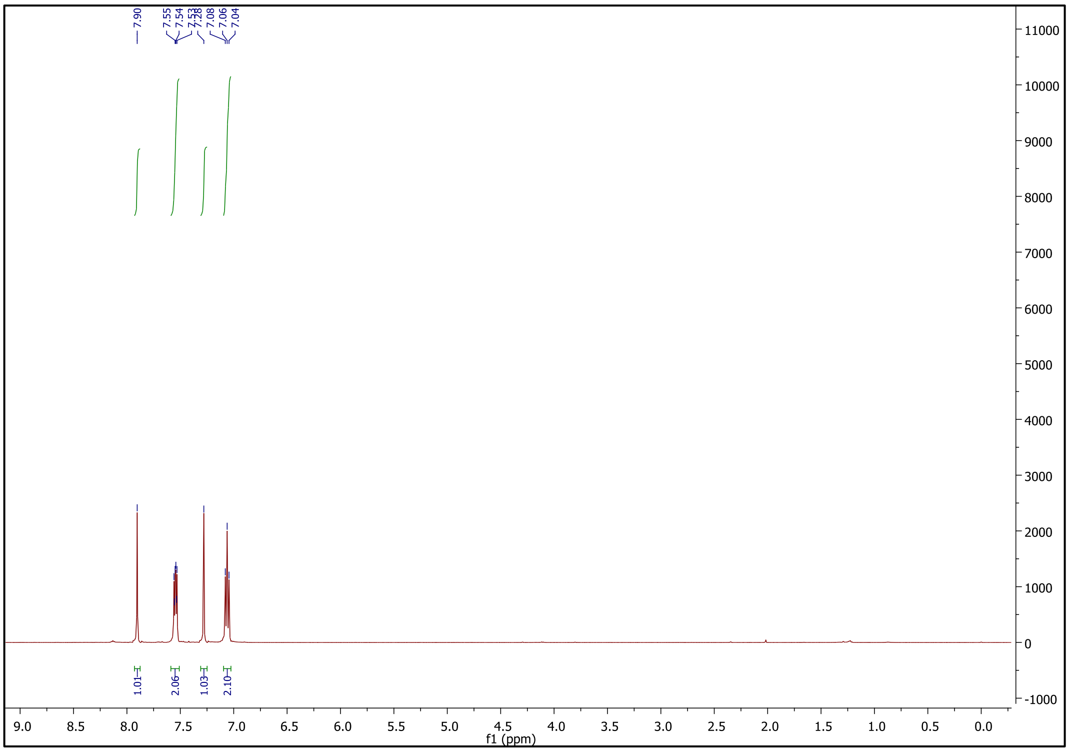

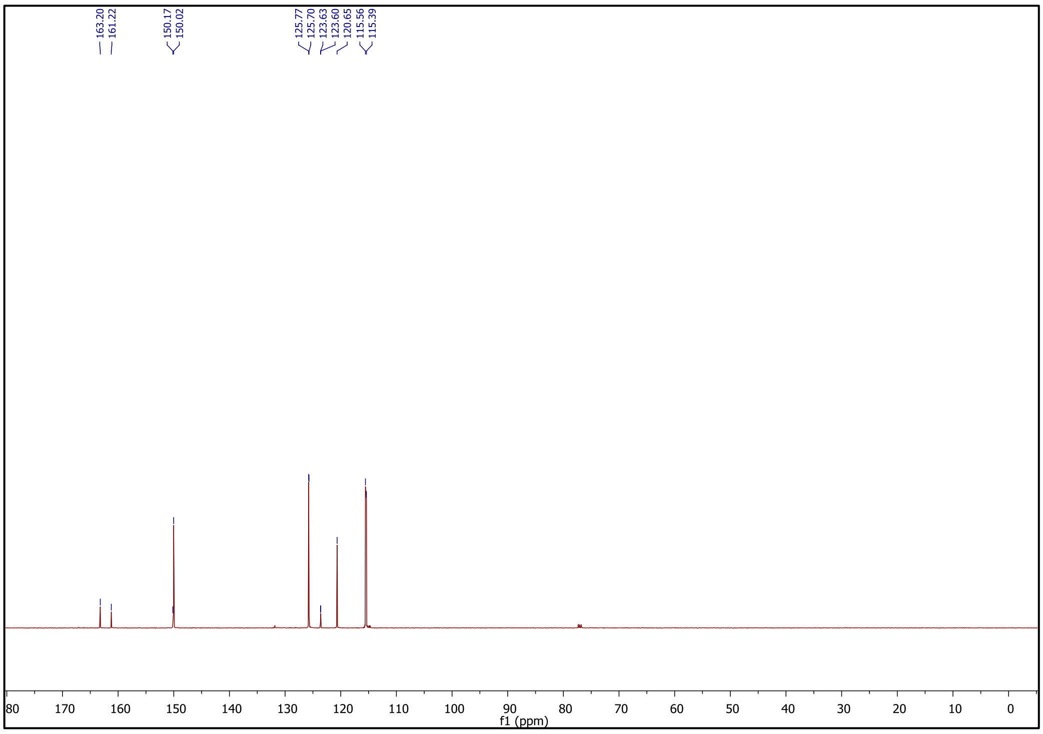

5-(3-(4,4,5,5-tetramethyl-1,3,2-dioxaborolan-2-yl)phenyl)oxazole (10):

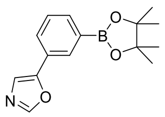

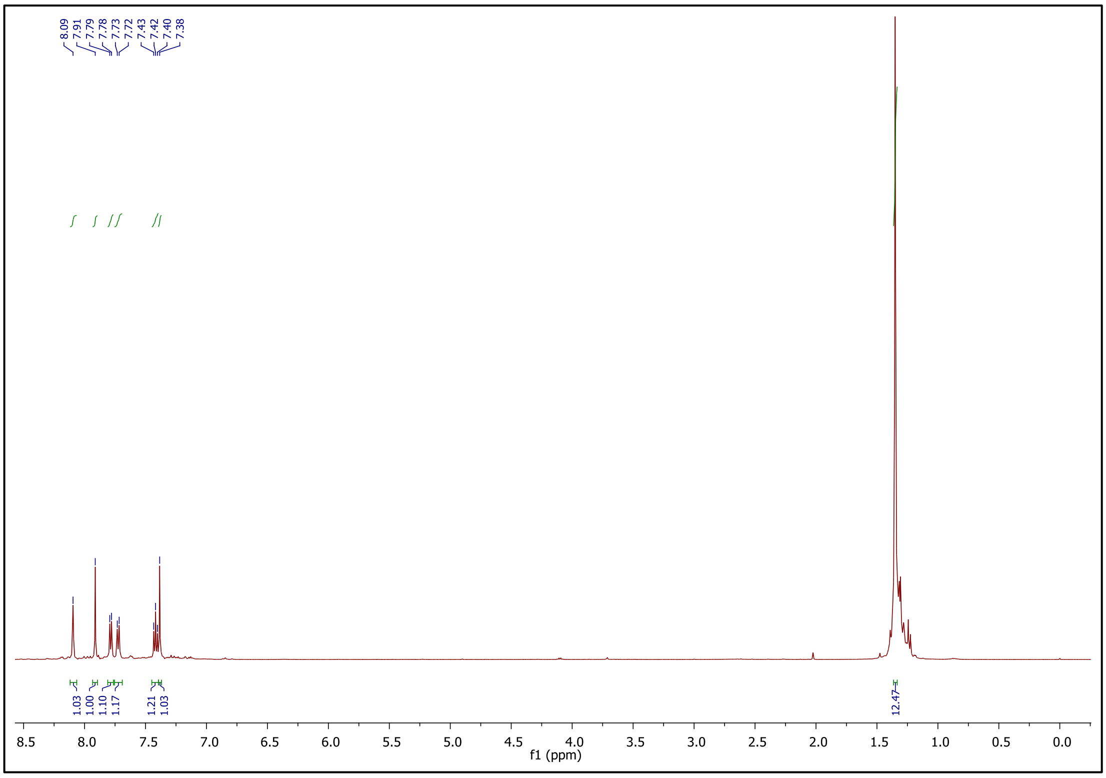

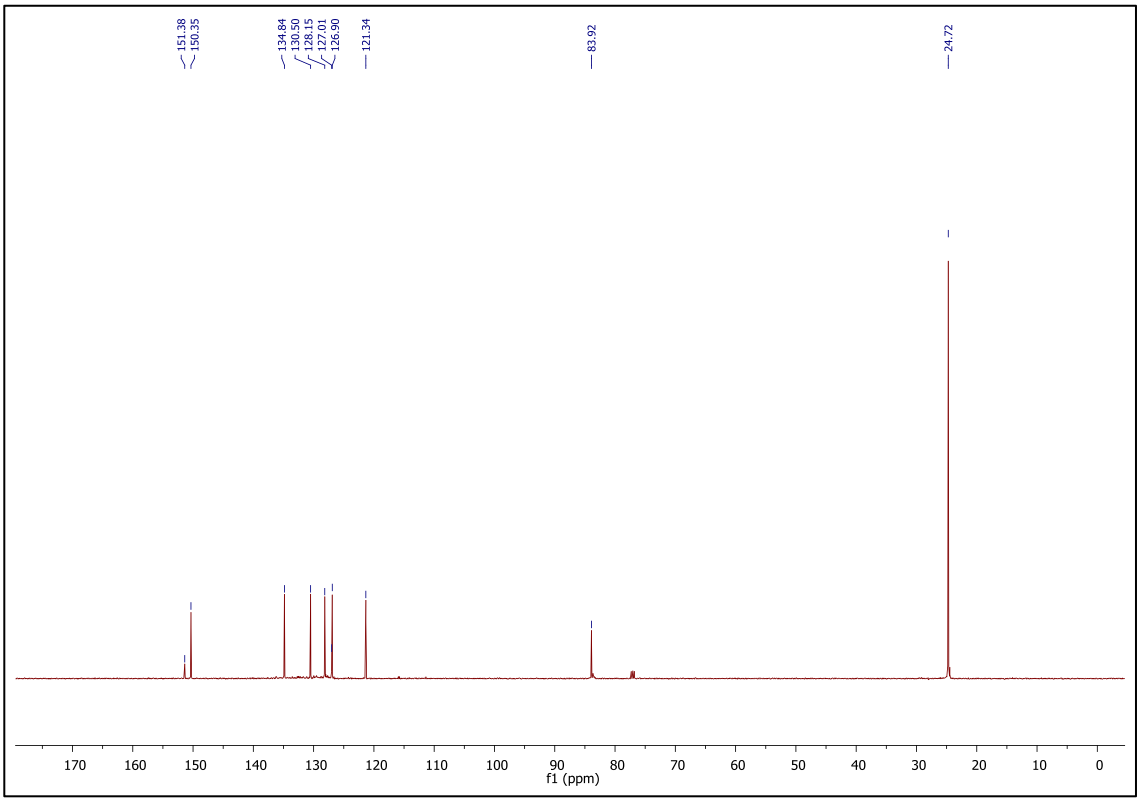

5-(3-fluorophenyl)oxazole ([19F]10):

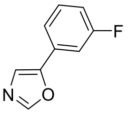

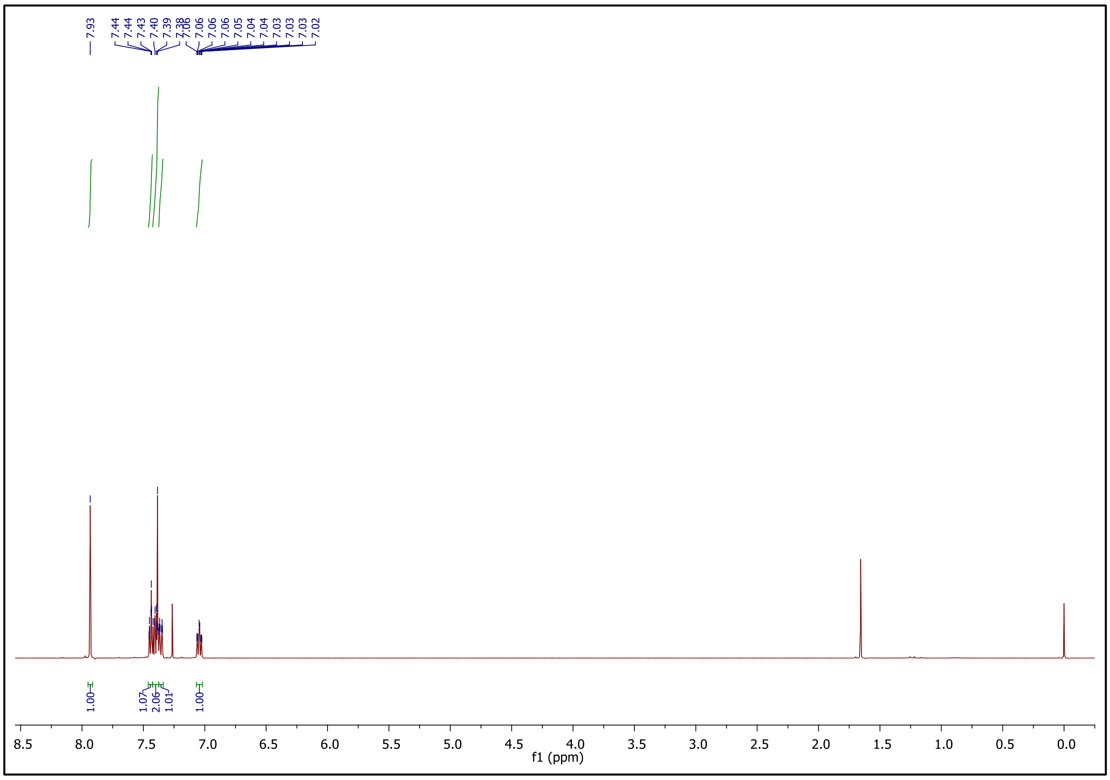

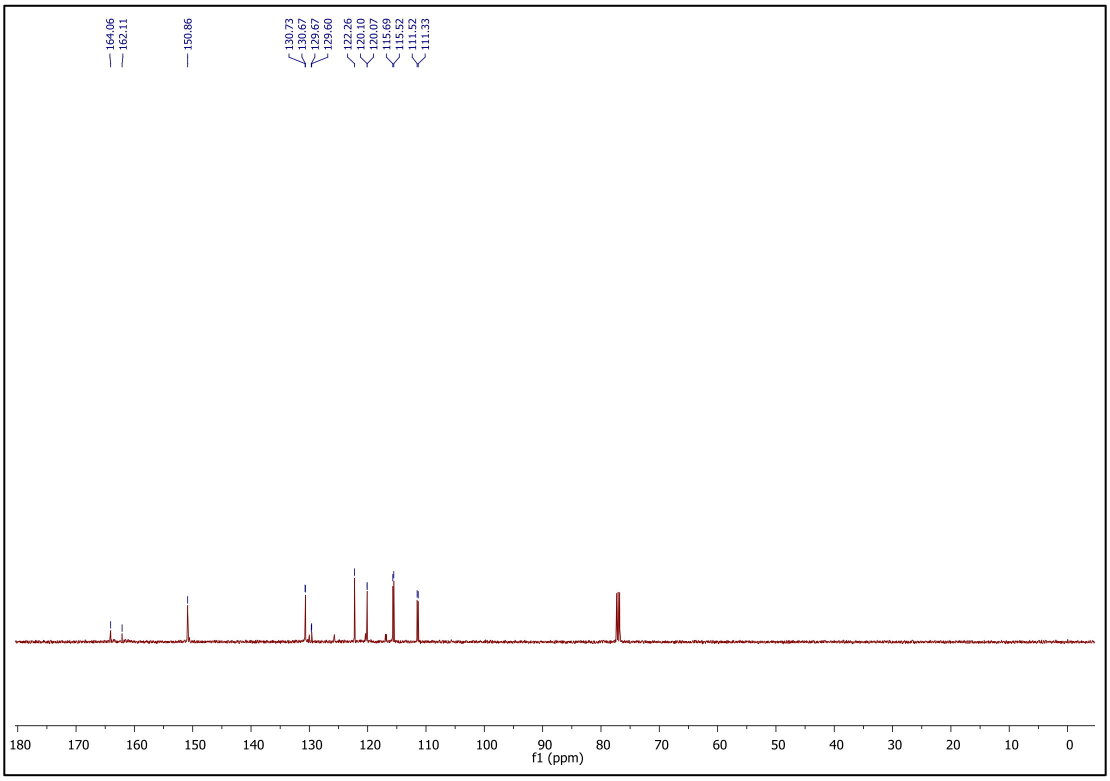

2-oxo-1-(4-(4,4,5,5-tetramethyl-1,3,2-dioxaborolan-2-yl)phenyl)-2-((3,4,5-trimethoxybenzyl)amino)ethyl 3-bromo-2-methylbenzoate (11):

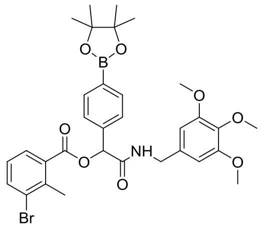

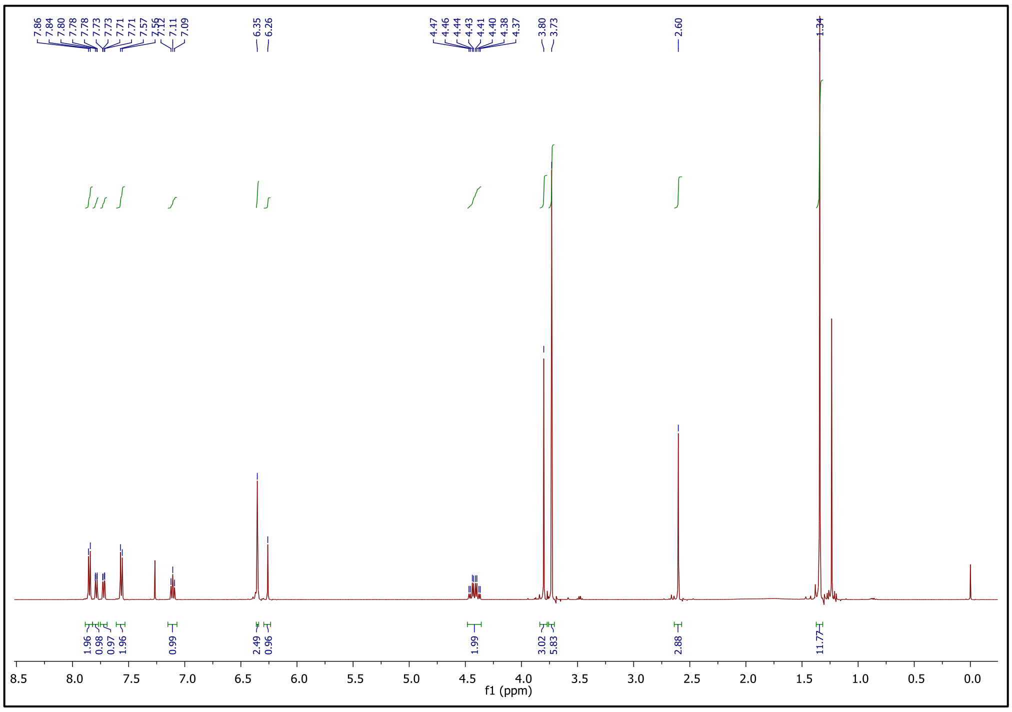

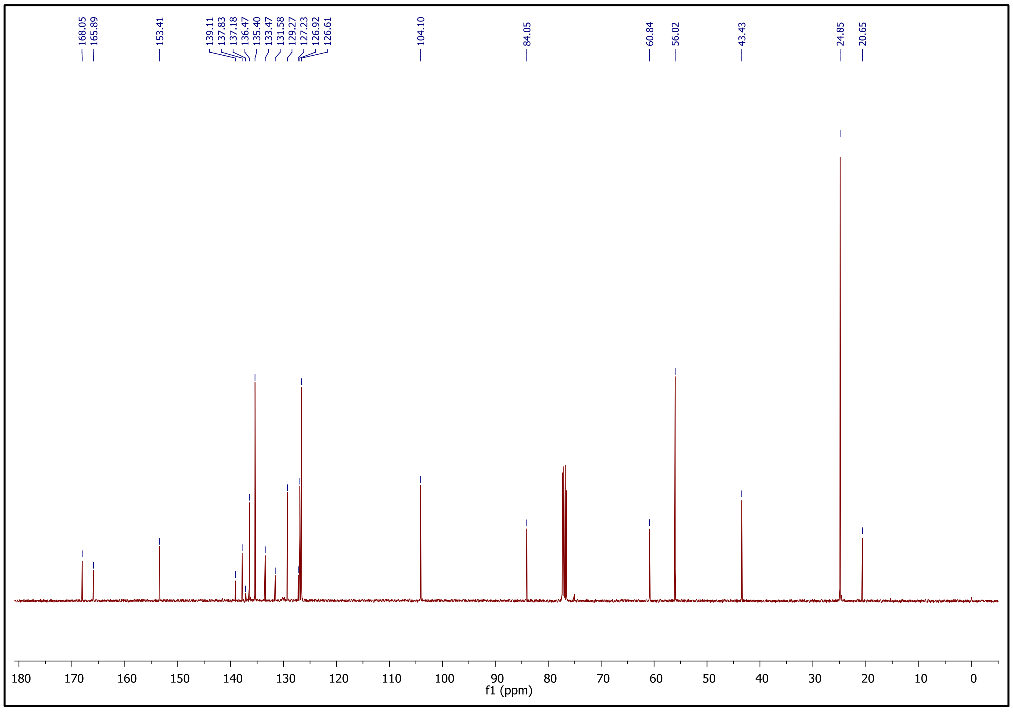

1-(4-fluorophenyl)-2-oxo-2-((3,4,5-trimethoxybenzyl)amino)ethyl 3-bromo-2-methylbenzoate ([^{19}F]11):

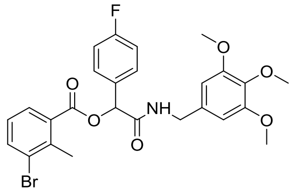

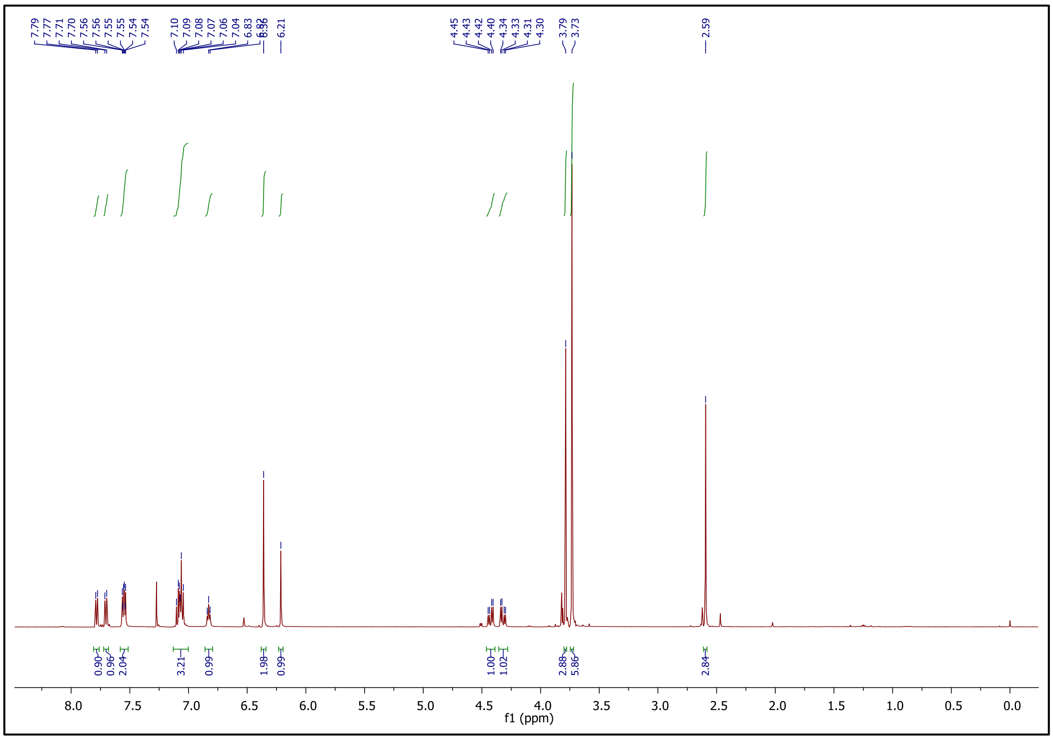

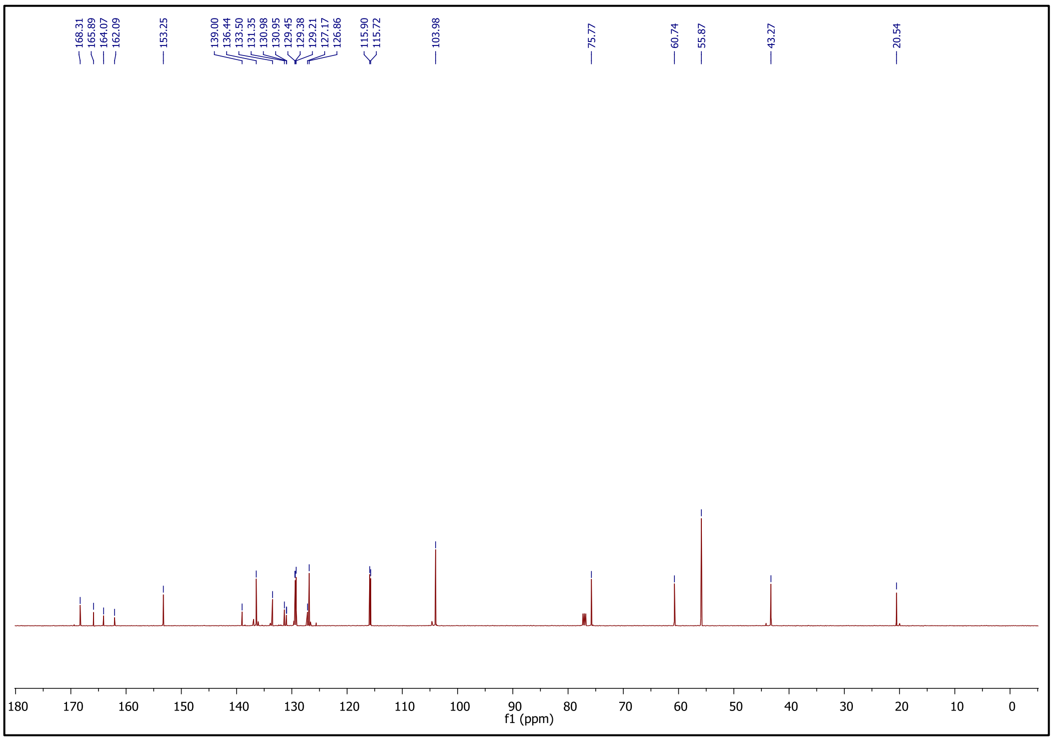

2-((benzo[d][1,3]dioxol-5-ylmethyl)amino)-2-oxo-1-(4-(4,4,5,5-tetramethyl-1,3,2-dioxaborolan-2-yl)phenyl)ethyl 2,4-dimethylbenzoate (12):

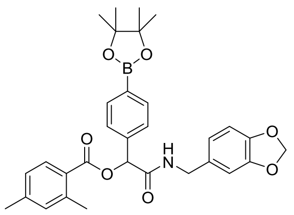

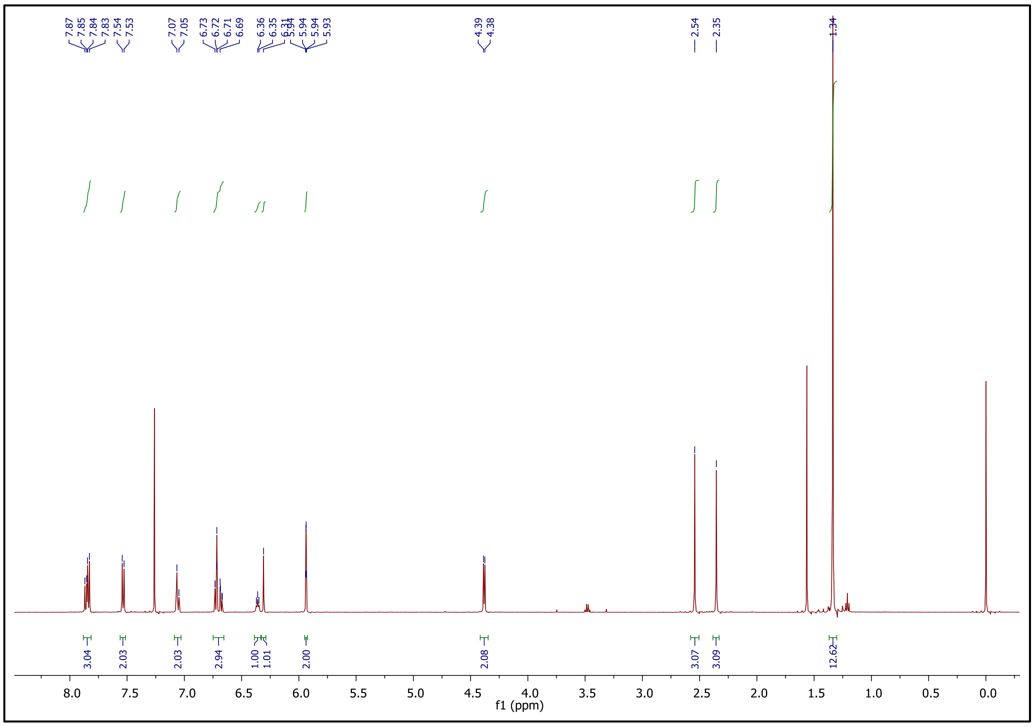

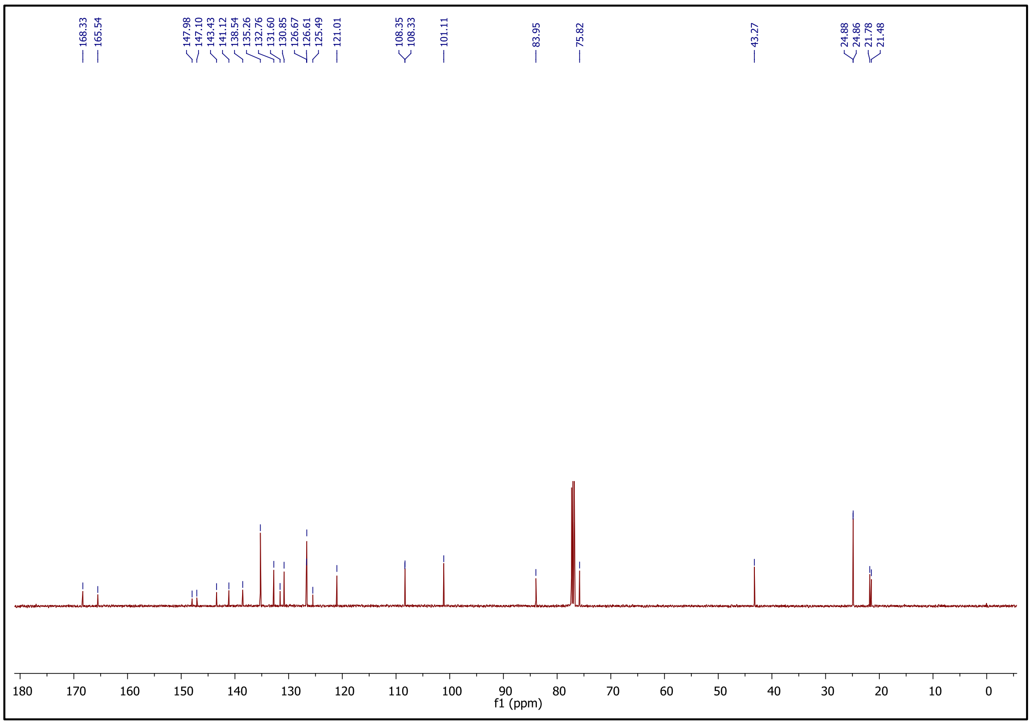

2-((benzo[d][1,3]dioxol-5-ylmethyl)amino)-1-(4-fluorophenyl)-2-oxoethyl 2,4-dimethylbenzoate ([^{19}F]12):

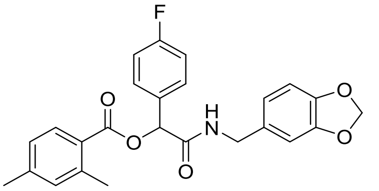

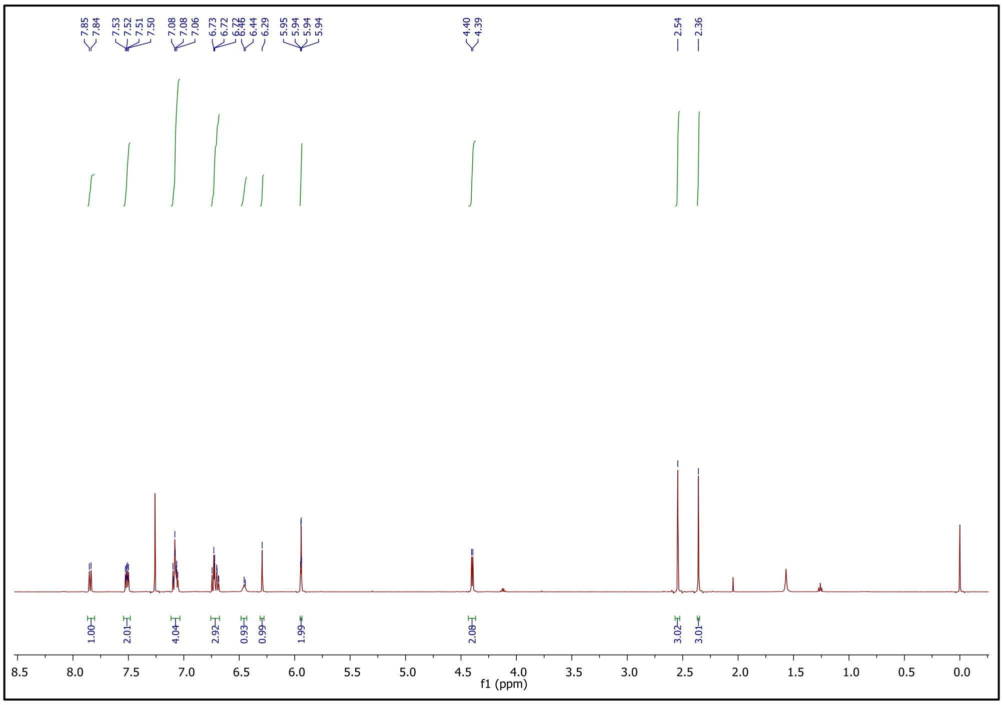

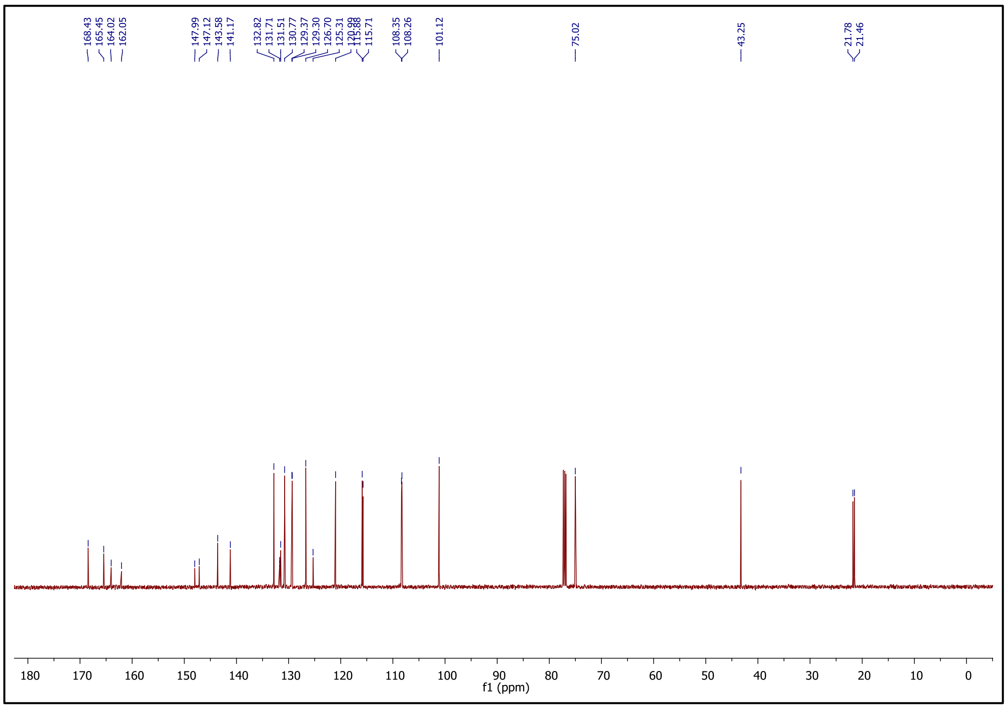

2-(benzylamino)-2-oxo-1-(4-(4,4,5,5-tetramethyl-1,3,2-dioxaborolan-2-yl)phenyl)ethyl cyclohexanecarboxylate (13):

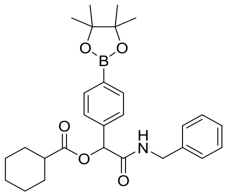

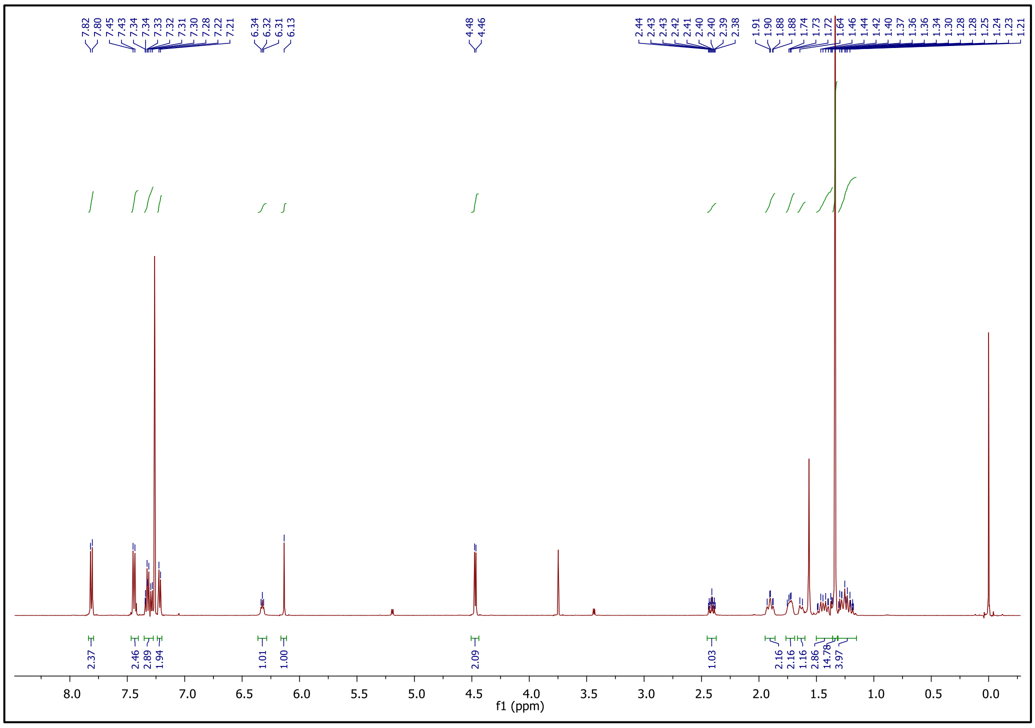

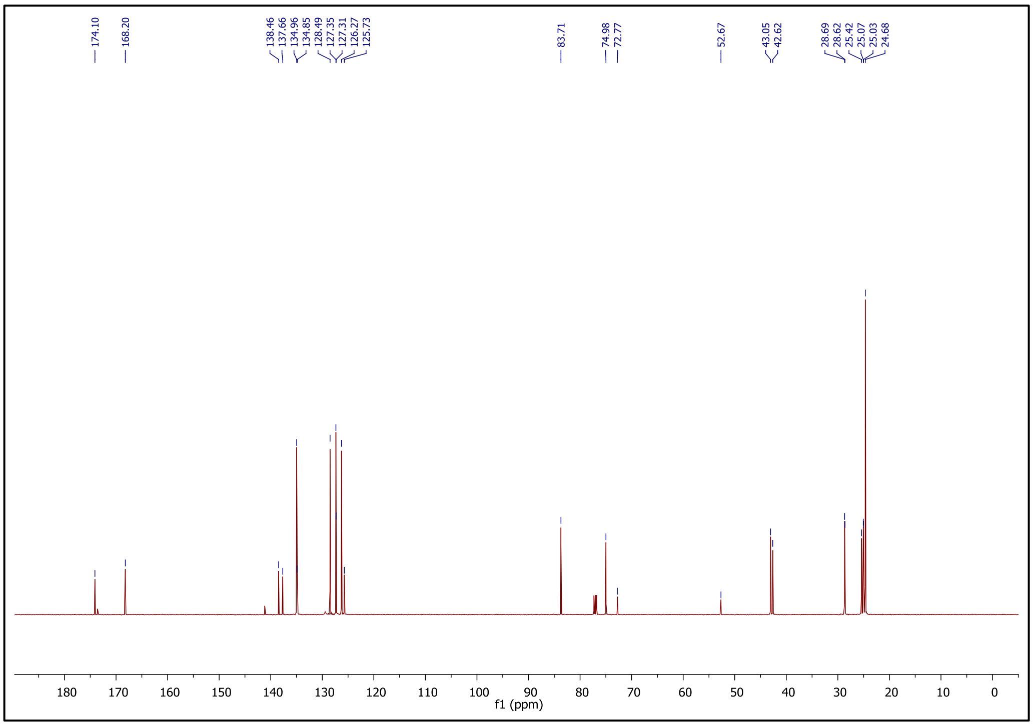

2-(benzylamino)-1-(4-fluorophenyl)-2-oxoethyl cyclohexanecarboxylate ([^{19}F]13):

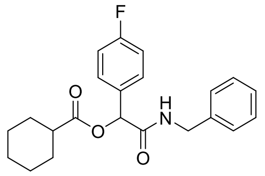

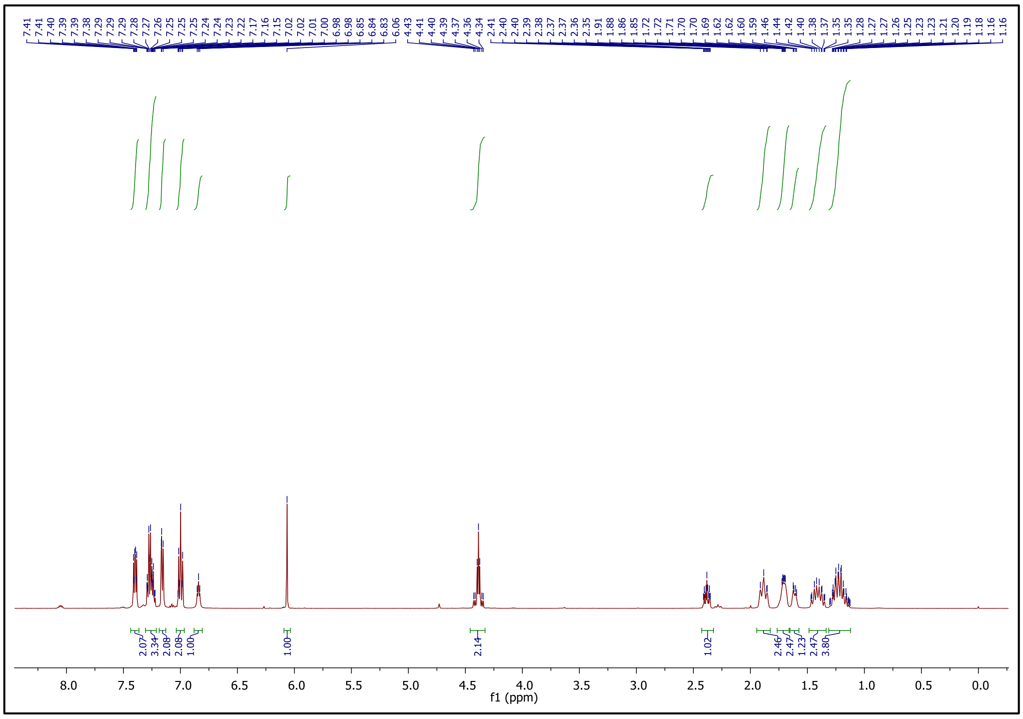

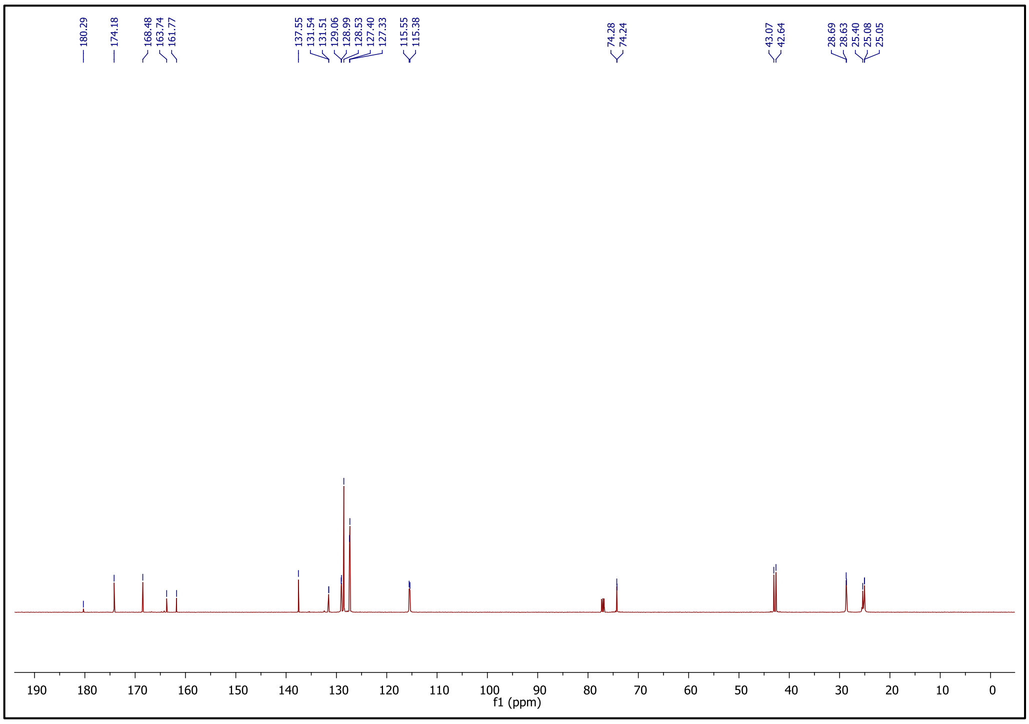

Supplement: Supplementary file 1 [file molecules-24-01327-s001.pdf]
